# Comparative Genome Analysis of *Campylobacter fetus* Subspecies Revealed Horizontally Acquired Genetic Elements Important for Virulence and Niche Specificity

**DOI:** 10.1371/journal.pone.0085491

**Published:** 2014-01-09

**Authors:** Sabine Kienesberger, Hanna Sprenger, Stella Wolfgruber, Bettina Halwachs, Gerhard G. Thallinger, Guillermo I. Perez-Perez, Martin J. Blaser, Ellen L. Zechner, Gregor Gorkiewicz

**Affiliations:** 1 Institute of Molecular Biosciences, University of Graz, Graz, Austria; 2 Department of Medicine, NYU Langone Medical Center, New York, New York, United States of America; 3 Department of Microbiology, NYU Langone Medical Center, New York, New York, United States of America; 4 Medical Service, VA New York Harbor Healthcare System, New York, New York, United States of America; 5 Institute for Genomics and Bioinformatics, Graz University of Technology, Graz, Austria; 6 Core Facility Bioinformatics, Austrian Centre of Industrial Biotechnology, Graz, Austria; 7 Institute of Pathology, Medical University of Graz, Graz, Austria; Free University of Berlin, Germany

## Abstract

*Campylobacter fetus* are important animal and human pathogens and the two major subspecies differ strikingly in pathogenicity. *C. fetus* subsp. *venerealis* is highly niche-adapted, mainly infecting the genital tract of cattle. *C. fetus* subsp. *fetus* has a wider host-range, colonizing the genital- and intestinal-tract of animals and humans. We report the complete genomic sequence of *C. fetus* subsp. *venerealis* 84-112 and comparisons to the genome of *C. fetus* subsp. *fetus* 82-40. Functional analysis of genes predicted to be involved in *C. fetus* virulence was performed. The two subspecies are highly syntenic with 92% sequence identity but *C. fetus* subsp. *venerealis* has a larger genome and an extra-chromosomal element. Aside from apparent gene transfer agents and hypothetical proteins, the unique genes in both subspecies comprise two known functional groups: lipopolysaccharide production, and type IV secretion machineries. Analyses of lipopolysaccharide-biosynthesis genes in *C. fetus* isolates showed linkage to particular pathotypes, and mutational inactivation demonstrated their roles in regulating virulence and host range. The comparative analysis presented here broadens knowledge of the genomic basis of *C. fetus* pathogenesis and host specificity. It further highlights the importance of surface-exposed structures to *C. fetus* pathogenicity and demonstrates how evolutionary forces optimize the fitness and host-adaptation of these pathogens.

## Introduction

The ε-proteobacterial genus *Campylobacter* comprises bacteria with a high degree of niche adaptation and host tropism [1]. The species colonize mucosal surfaces and are animal and human pathogens [2]. The genomes of *Campylobacter* spp. are not large (≈1.5 Mbp) and show characteristics of genome decay typical for niche-adapted bacteria [3]. These features make *Campylobacter* species ideal model systems to study genetic contributions to niche specificity and virulence by comparative genome analysis [3]. Multi locus sequence typing (MLST) has shown that the two *C. fetus* subspecies, *C. fetus* subsp. *fetus* and *C. fetus* subsp. *venerealis*, have a clonal population structure [4] and differentiation of the taxa is only partially successful [5]. Both subspecies are important veterinary pathogens causing abortions and infertility in ruminants [6]. *C. fetus* subsp. *venerealis* is a bovine-adapted “clone” [7] causing venereal infections and epidemic abortion in cattle. Statutory preclusion of *C. fetus* subsp. *venerealis* infection underscores the importance of this veterinary pathogen [8], but human infections are rare [6]. In contrast the generalist subspecies, *C. fetus* subsp. *fetus*, colonizes the intestinal and the genital-tract of multiple hosts including sheep, cattle, birds and humans. It is an emerging human pathogen, leading to invasive infections and even death [9], [10]. Most bacteremic illnesses caused by *Campylobacter* are due to *C. fetus* [9], [11].

*C. fetus* displays two major (O-antigen based) sero-types, A and B, and a rare variant AB [12]. The sero-types correlate with the type of surface array protein (Sap) expressed by the bacterium [13] and differ in their lipopolysaccharide (LPS) composition [12], [14]. The Sap-layer (S-layer) creates a paracrystalline proteinaceous cover enabling *C. fetus* to resist serum bactericidal activity, and by phase variation to overcome immune recognition [11], [15], [16]. Sero-type A strains expressing SapA are more frequently isolated from human blood than sero-type B strains expressing SapB. The cattle-adapted *C. fetus* subsp. *venerealis* is exclusively sero−/sap-type A (type A). Four different *Campylobacter* clades were identified using MLST [4] and represent the genotypes (I) *C. fetus* subsp. *venerealis* type A, (II) *C. fetus* subsp. *fetus* type A or (III) type B and (IV) reptile *C. fetus* type A. The reptilian clade diverges most substantially from the other three closely related genotypes.

The evolutionary interplay between microbial pathogens and their hosts is a continual process of adaptation, manifested by genomic variation of host adaptation factors, and by the gain and loss of genes via horizontal gene transfer (HGT). The underlying hypothesis for this study was that genome reduction and acquisition of relatively few novel genes has enabled *C. fetus* to adopt distinct subspecies-specific lifestyles. To evaluate this, we performed comparative genetic analyses of *C. fetus* subsp. *venerealis* (type A) and *C. fetus* subsp. *fetus* (type A), and we compared regions of the two type A strains to type B and reptile *C. fetus* strains. To gain initial insights into the transcriptional organization of *C. fetus*, differential RNA-sequencing (dRNA-seq) was performed with the sequenced strains of both subspecies. The analyses revealed many of the molecular details involved in (sub)speciation and virulence of *C. fetus* and explain the strikingly different host tropism and clinical manifestations of these pathogens.

## Results

### Comparative Genomics of *C. fetus* Subspecies

The genome of the bovine strain *C. fetus* subsp. *venerealis* 84-112 (type A) was sequenced generating 216.8 Mbp sequence data (≈112-fold coverage). This strain harbors a single circular chromosome 1.93 Mbp in size with GC-content of 33.3% and a circular extra-chromosomal element of 61,141 bp with GC-content of 31.5%. Until now, the only other closed *C. fetus* genome publicly available was the human isolate *C. fetus* subsp. *fetus* 82-40 (type A). That 1.77 Mbp genome also has GC-content of 33.3%. Analysis of the two genomes revealed that they are highly syntenic with 92.9% overall sequence identity. The homologous regions exhibit 99.8% DNA identity. 180 kbp were unique for strain 84-112 and 35 kbp of unique sequences were identified in strain 82-40. Including the 73 extra-chromosomal element open reading frames (*orf*s), strain 84-112 harbors 204 unique *orf*s. Nearly all represent putative type IV secretion system (T4SS) components, transposons, or hypothetical proteins **(File S4)**. The 25 *orf*s unique for strain 82-40 encode putative CRISPR associated (Cas)-proteins, LPS-biosynthetic enzymes, or hypothetical proteins **(File S4)**. General genomic characteristics are summarized in **Table 1**.

**Table 1:** *C. fetus* genome attributes, including the extra-chromosomal element.

Attributes	*Cff*	*Cfv*	*Cfv*
	strain 82-40	strain 84-112	ICE_84-112
*Genome size (bp)	1,773,615	1,926,886	61,141
*GC-content %	33.31	33.34	31.54
*coding DNA sequence (# of *orfs*)	1,769	1,992	73
*rRNA genes	6	6	–
*tRNA genes	43	43	–
***Genomic Islands***	2 (FGI I–II)	4 (VGI I–IV)	–
***T4SS loci***			
* tra*-like gene cluster	0	0	1
* vir*-like gene cluster	0	2	1
***Flexible gene pool***			
Integrase XERCD family	1	1	0
Integrases/recombinases	1	2	0
Insertion Elements (# of copies)	0	ISHa1152 (2)	ISHa1152 (3)
	0	ISC1904 (3)	0
Prophage-like gene clusters	1	3	0
***CRISPR***			
Spacers (# of copies)	21 (1)	24 (1)	0
	26 (1)		
* cas*-genes	*cas1-6*	0	0

according to RAST annotation.

**Figure 1:**
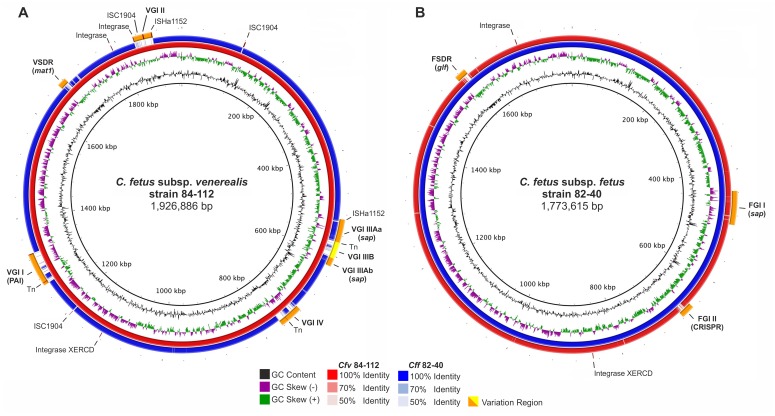
Genome comparisons of *C. fetus* subspecies. Plots were generated using *C. fetus* subsp. *venerealis* 84-112 (*Cfv*) as a reference (**A**) or *C. fetus* subsp. *fetus* 82-40 (*Cff*) (**B**). Inside tracks represent GC-content (ring 1) and GC-skew (ring 2). *Cff* is shown in blue and *Cfv* in red. Variation regions (VR) relative to the reference genome are indicated in orange/yellow and named according to the corresponding Genomic Island (GI) or the subspecies definition region (SDR). (V) and (F) in the feature names designate the subspecies *venerealis* and *fetus*, respectively. Important genes or features are indicated in parenthesis. Positions of selected mobility genes are indicated.

**Figure 2:**
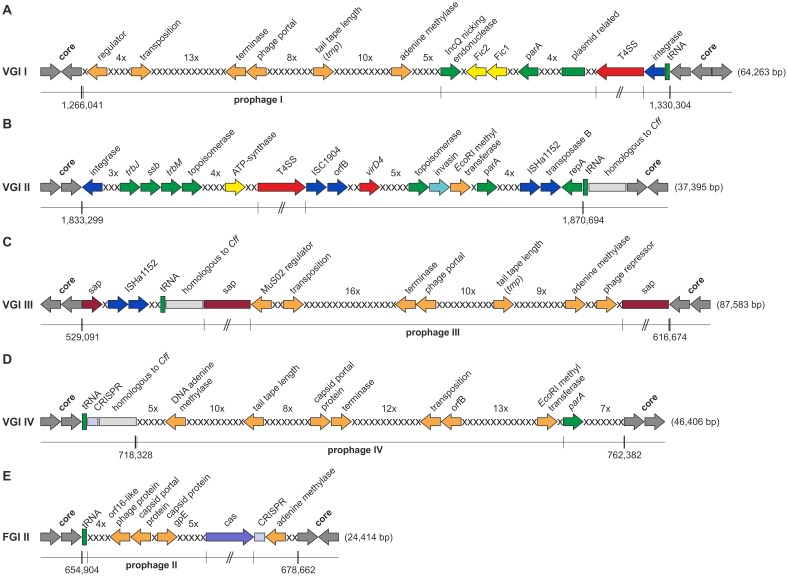
Comparative overview of Genomic Islands (GIs). (**A**) VGI I (PAI) with the T4SS and putative prohage I, (**B**) VGI II with a *vir*-gene cluster and plasmid-related genes, (**C**) VGI III containing the surface array protein cluster and prohage III, (**D**) VGI IV containing the CRISPR-array and prophage IV and (**E**) FGI II with prophage-related genes (prophage II) and the CRISPR-cluster (array and *cas*-genes). The GI borders to genes shared between the subspecies (grey) are indicated with nucleotide position. Gene clusters are colored as follows: phage-related genes (orange), plasmid related genes (green), integrases and transposases (blue), T4SS (red), effector proteins (yellow), surface array proteins (purple), *cas*-genes (lavender), tRNAs (green boxes); Each x represents a hypothetical protein and their numbers in tandem are indicated above.

Comparative genome plots clearly illustrate that the unique DNA stretches are located in distinct genomic regions (termed variation regions, VR) scattered across the syntenic genomic core **(** **Figure 1** **)**. *C. fetus* subsp. *venerealis* 84-112 harbors 5 VRs and *C. fetus* subsp. *fetus* 82-40 harbors 3 VRs **(** **Figure 1** **, Table S1 in File S5)**. All of these regions have features indicative of horizontal acquisition including a shift in %GC-content compared to the core genome, the presence of mobility-related genes (e.g. prophages, transposases) or proximity to tRNA genes, presumably marking their insertion sites into the chromosomal backbone. Two of the VRs of strain 84-112, Venerealis Genomic Island (VGI) I and VGI II, have no counterpart in strain 82-40. Two other VRs are shared between the two subspecies. VGI III of strain 84-112 corresponds to the position of Fetus Genomic Island (FGI) I of strain 82-40. The position of VGI IV corresponds to FGI II. The respective regions of variation are not identical between the two subspecies, but are highly similar, suggesting a common origin. Notably, the VRs of strain 84-112 carry additional blocks of genes, which are predominantly prophage-related. The extra insertions appear to interrupt functional gene modules, thus VGI III can be divided into three subsections designated VGI IIIA-1, VGI IIIA-2, and VGI IIIB (see below, **Figure 2C** **, Table S1 in File S5**). In strain 84-112, phage-related genes or transposases flank the VGIs. Similar genes are absent in strain 82-40 except for one area on FGI II containing prophage-like features (see below, **Figure 2E**).

One VR region **(** **Figure 1** **, Figure S1)** that co-localizes in the genomes of both subspecies was designated as the Venerealis- or Fetus Subspecies Definition Region (VSDR/FSDR). These regions are marked by comparatively low GC-content (30.7% and 29.4%, respectively). They contain genes putatively involved in surface carbohydrate metabolism as analyzed below and differentiate the two subspecies.

Metabolic reconstruction based on the genome data and comparative analyses of metabolic pathways using RAST and SEED revealed only two differences between the genomes. *C. fetus* subsp. *fetus* 82-40 harbors two *orf*s putatively involved in thiamin (vitamin B1) biosynthesis, namely a phosphomethylpyrimidine kinase (EC 2.7.4.7) and the thiamin biosynthesis protein ThiC (peg.404), which are absent from the genome of *C. fetus* subsp. *venerealis* 84-112. ThiC does not appear to be specific for *C. fetus* subsp. *fetus*, however, since a *thiC* homolog is also present in the unfinished genome of *C. fetus* subsp. *venerealis* NCTC 10354. Also no other obvious differences in respiration systems, nutrient transporters and catabolic or anabolic pathways were identified. Whether more subtle genetic differences, like insertions, point mutations or variation in transcriptional control, which might influence metabolism, contribute to the different biology of *C. fetus* subspecies remains to be elucidated.

In summary, comparative genomics revealed that the two *C. fetus* subspecies are highly syntenic, but the chromosome of *C. fetus* subsp. *venerealis* 84-112 is about 9% larger. The genomic VRs distinguishing the two subspecies are located within a small number of hot-spots, displaying features typical for horizontally acquired DNA.

### VGI I and II Contain T4SS-related Genes, Prophage- and Plasmid-like Features

We previously identified and characterized a pathogenicity island (PAI) in *C. fetus* subsp. *venerealis* that was absent in all 45 *C. fetus* subsp. *fetus* isolates tested [17]. The PAI contained a full set of *virB*/*virD4* genes prototypical for a T4SS (for review see [18]). The T4SS of strains ATCC 19438 and 84-112 mediate conjugative DNA transfer as well as host interaction [17], [19]. This PAI is located in VGI I of strain 84-112 **(** **Figure 2A** **)**. VGI I also harbors the putative prophage I encompassing a region of 33.7 kb (position: 1,266,041 to 1,299,761) with 47 *orfs* and a GC-content of 35.4%.

The gene organization of VGI II is less consistent, but with conserved functional modules **(** **Figure 2B** **)**. Although T4SS-related genes are present, the system lacks *virB5* and *virB6* and may be non-functional (see below, and **Figure S3**). Under laboratory conditions, we did not detect transcription of these genes (data not shown). The gene for transposase ISHa1152 suggests a putative integration site for VGI.

### FGI I and VGI III Contain the *Sap*-locus

**Figure 3:**
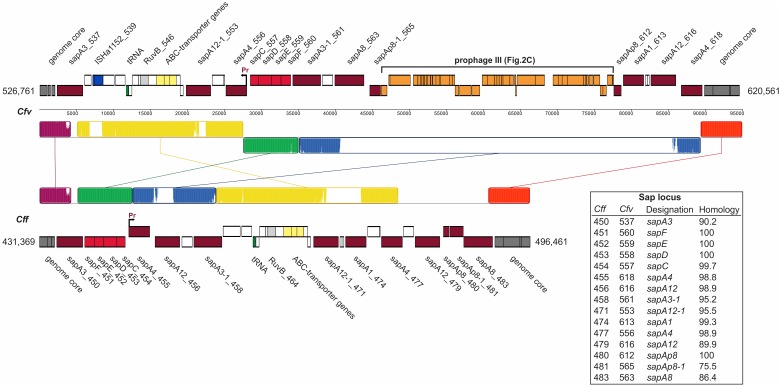
Schematic representation and structural comparison of VGI III and FGI I (sap region). MAUVE was used to compare the VRs of both subspecies for visualization of rearrangements and insertions. Regions free of rearrangements are indicated by colored colinear blocks. White color within these blocks indicates insertions or non-homologous regions. Important *orfs* are colored and labeled. S-layer genes (purple) were identified in both *C. fetus* strains. The *sap*-promoter is indicated. In *C. fetus* subsp. *venerealis* 84-112, the *sap* genes were disrupted by an inserted prophage (orange). White boxes are mainly hypothetical proteins. Detailed annotation information can be found in File S1. Genes are labeled with RAST-peg numbers and the inset table lists homologous *sap* genes of the subspecies.

**Figure 4:**
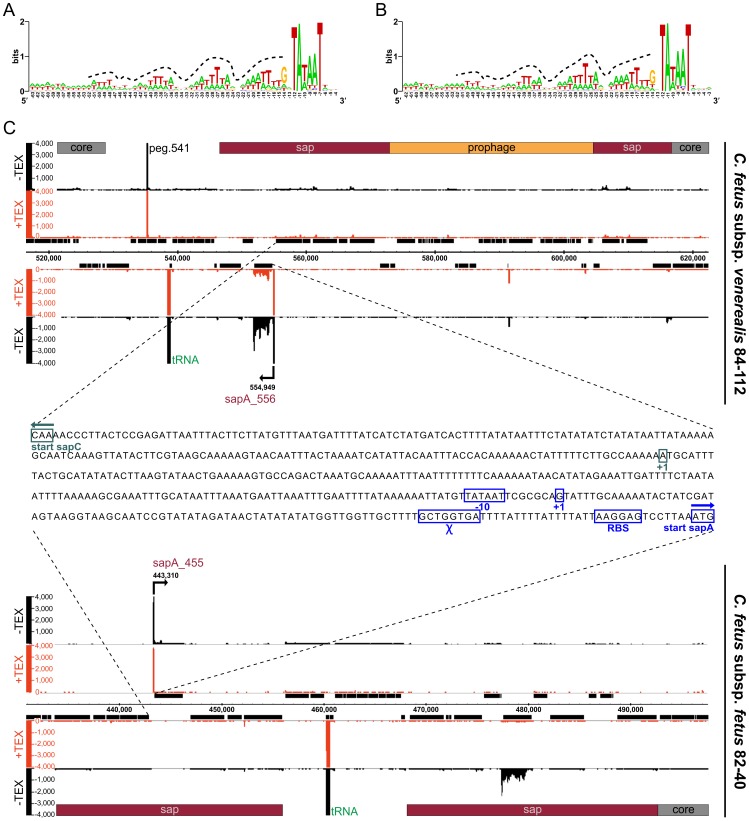
*C. fetus* promoter sequence and transcriptional organization of the *sap*-locus. Promoter consensus sequence for (**A**) *C. fetus* subsp. *venerealis* 84-112 (*Cfv*) and (**B**) *C. fetus* subsp. *fetus* 82-40 (*Cff*). The promoter motif is defined by an extended Pribnow box (tgnTAtaAT) at the −10 position. The −35 motif is replaced by a periodic AT-rich signal upstream of position −14 (dotted line). (**C**) Transcriptional organization of *Cfv* VG III **(top)** and *Cff* FGI I **(bottom),** identical *sap*-promoter sequence of *Cfv* and *Cff* **(middle)**.

The *sap*-locus of *C. fetus* is present in both subspecies and represents the best-characterized *C. fetus* virulence attributes [11], [15], [16]. In *C. fetus* subsp. *fetus* 82-40 the *sap* genes are located on FGI I close to a tRNA and putative ABC-transporter genes **(** **Figure 3** **)**. In *C. fetus* subsp. *venerealis* 84-112, the comparable region of VGI III is highly similar to FGI I. However, a block of phage-related genes and a series of genes for hypothetical proteins indicate the presence of another prophage **(** **Figure 2C** **,** **Figure 3** **)** apparently leading to rearrangement and separation of the *sap* genes that may affect S-layer variation of *C. fetus* subsp. *venerealis* 84-112. The transcriptome analysis indicates that the insertion of prophage III did not lead to inactivation or truncation of sapAb8_612 **(** **Figure 4** **)**. As in VGI I, the ISHa1152 transposase gene was detected, putatively marking a site for extra-chromosomal DNA insertion.

### FGI II and VGI IV Contain CRISPR Loci

We identified CRISPR-repeats on the genomes of both *C. fetus* subspecies **(Figure S1)**. In *C. fetus* subsp. *venerealis* 84-112, a single locus (nt 684,618 to 686,228) (Cfv_CRISPR) displays the typical features of a CRISPR-array with 30-bp direct repeats (DR), separated by 21 different spacers. No *cas*-homologues were identified. Two CRISPR-arrays (nt 655,350 to 656,762 and nt 674,442 to 676,187) were identified in *C. fetus* subsp. *fetus* 82-40 (Cff_CRISPR_1 and Cff_CRISPR_2), but only Cff_CRISPR_2 is in close proximity to *cas*-gene homologues. The DRs and the leader sequence are identical in both subspecies. Some spacers are shared between Cfv_CRISPR and Cff_CRISPR_1, but Cff_CRISPR_2 has no homology to Cfv_CRISPR and Cff_CRISPR_1. Sequences homologous to the spacers of the CRISPR loci were not detected in public DNA databases, thus their putative DNA targets remain unknown.

Since *Cas1* is a hallmark of dynamic CRISPR arrays, we screened 102 *C. fetus* strains for its presence. *Cas1* was detected in 19 (47.5%) of 40 subsp. *fetus* subsp. *fetus* isolates but was absent in all 62 subsp. *venerealis* isolates (Odd ratio = 110, 95% CI: 6.3 to 1,897, p = 0.0012). In strain 84-112 another prophage-like gene cluster (prophage IV) is present instead of the *cas*-genes and the second CRISPR array **(** **Figure 2D** **, Figure S1)**. Interestingly, type B strains are more likely to carry the *cas1* gene (14 of 15) compared to type A strains (5 of 24) (Odd ratio = 53.2, 95% CI: 5.6 to 507.4; p = 0.0006) **(Table S6 in File S5)**.

### The Extra-chromosomal Element of *C. fetus* subsp. *Venerealis* 84-112 Displays Features Typical for Integrative Conjugative Elements (ICE)

The extra-chromosomal element was designated as ICE_84-112 and is the first ICE described in *C. fetus* (physical map **Figure S2**; annotation details in **File S3**). Conjugative transfer (*tra*) and other genes of apparent plasmid origin were identified but autonomous replication features were lacking. The T4SS locus, termed ICE_*trb/tra,* most likely is involved in horizontal self-transfer, based on its close relation to the broadly disseminated RP4-like systems. Several phage-related genes and transposases, including the ISHa1152 transposase, could aid chromosomal integration and excision of the ICE **(** **Figure 1A** **, Figure 2BC)**. A region with structural homology to the PAI of VGI I was identified on ICE_84-112 (termed ICE_*vir*). ICE_84-112 also encodes proteins with a domain called filamentation-induced by cyclic AMP (Fic). This domain is similarly present in Fic1 and Fic2 expressed by the PAI of VGI I [17], [19]. We screened our *C. fetus* collection for the presence of ICE_84-112 using the ICE specific genes *fic3* and *fic4* as PCR targets. Of 62 *C. fetus* subsp. *venerealis* strains, 7 harbored the ICE-related genes **(Table S6 in File S5)**. The target genes *fic3* and *fic4* were not detected in any of the 40 *C. fetus* subsp. *fetus* strains tested. Transcriptome analysis showed expression of the majority of genes on ICE_84-112.

ICE_84-112 may replicate extra-chromosomally via a conjugative transfer replication mode, as proposed for other ICEs [20], [21], since the obligatory features including a putative IncP*_nic_*-site, an *origin of transfer*-binding protein, a relaxase, a helicase and a nicking-endonuclease were identified **(Figure S2)**. According to the classification of Barcillán-Barica *et al*. [22], the putative ICE_84-112 (CDS peg.24) relaxase belongs to the MPB_P1_ group (clade MOB_P11_) of relaxases, displaying the typical conserved sequence motifs. Most of the MPB_P1_ group of relaxases are linked to conjugative plasmids. Lee *et al*. [20] demonstrated that the chromosomally encoded *Bacillus subtilis* helicase PcrA associates with ICE*Bs1* during replication. ICE*Bs1* is defective for replication in *pcrA*-mutant strains and *pcrA* is necessary for ICE*Bs1* conjugation. PcrA orthologs, which could be recruited for replication and conjugation, are present in both *C. fetus* subspecies (84-112 CDS peg.56 & peg.1280 and 82-40 CDS peg.690 & peg.934).

### dRNA-seq Identified Transcriptional Start Sites and the Typical Promoter Structure for Campylobacterales in Both Subspecies

Transcriptional start sites **(**TSS) annotation, performed computationally, allowed classification of TSS according to their location relative to the surrounding *orfs*. The analysis revealed a variety of transcripts with TSS located upstream and internal to their respective *orf* but also included antisense transcripts. Many TSS were simultaneously assigned to more than one category **(Figure S5)**.

Sequences upstream of the annotated TSS were used to define *C. fetus* promoter motifs. *C. fetus* subsp. *venerealis* has more *orfs* than *C. fetus* subsp. *fetus* and we identified 797 promoter sequences in strain 84-112 and 575 promoter sequences in strain 82-40, with an extended Pribnow box (tgnTAtaAT) as the −10 motif in both subspecies. Consistent with other Campylobacterales [23], [24] the typical bacterial −35 motif is replaced by a periodic AT-rich signal upstream of position −14 **(Figure 4AB)**. This also is evident in the *sap*-locus located on genomic islands VGI III and FGI I. The intragenic promoter region between *sapC* (component of the Sap-transporter) and a respective *sap*-homologue is 100% conserved between the subspecies and only the *sap*-homolog directly downstream of the promoter is transcribed **(** **Figure 4C** **)**.

### *C. fetus* subsp. *Venerealis* 84-112 Harbors T4SS-related Loci

*C. fetus* subsp. *venerealis* 84-112 harbors four regions showing homology to T4SS genes **(Figure S3)**. Two are on the chromosome within VGI I (PAI) and II **(Figure 2AB)** and two are located on ICE_84-112 **(Figure S2)** annotated as ICE_*trb*/*tra* and ICE_*vir.* The ICE_*trb/tra* region differs from the other T4SS and shares homology to IncP plasmid RP4. For the ICE_*vir* region, blast searches and phylogenetic analyses using VirB4 and VirB11 [25] identified the PAI T4SS **(Table S2 in File S5)** and an as yet uncharacterized T4SS of *Campylobacter hominis* as their closest neighbor. The *vir*-genes located on VGI II did not share high homology with the *vir*-genes present on either VGI I or ICE_84-112. Instead the closest relative is a putative T4SS present in *C. rectus* RM3267, indicating a different origin. Finally, transcriptome analysis indicated that the VGI III T4SS components are not transcribed under laboratory conditions, whereas expression of the PAI T4SS (VGI I), ICE_*vir* and ICE_*trb/tra* was detected (data not shown, and [17]).

### Genes Involved in LPS-biosynthesis Distinguish *C. fetus* Sero−/Sap-types

The subspecies definition regions contain unique genes putatively involved in LPS-biosynthesis. Although inserted at the same chromosomal position in both subspecies **(Figure 1AB)** the islands display only limited similarity **(Figure S4)**. One obvious difference was that VSDR encodes a putative maltose O-acetyltransferase (*mat1*) (cd04647) and FSDR a putative UDP-galactopyranose mutase (*glf*) (EC 5.4.99.9) **(Figure S4)**. Remarkable is the low GC-content of the VSDR and FSDR of 30.7% and 29.4%, respectively **(Table S1 in File S5)** and the absence of tRNA or apparent mobility genes.

Acetyltransferases generally catalyze the CoA-dependent acetylation of the 6-hydroxyl group of sugar substrates. Maltose O-acetyltransferases exclusively acetylate maltose and glucose. *C. fetus* type A LPS contains 74.5% mannose as well as 6.5% D-glucose [26] and thus may serve as a substrate for Mat1. UDP-galactopyranose mutase (*glf*) drives the conversion of the ring form of galactose from pyranose to furanose. The latter isomer is specifically found in glycoconjugates (including LPS) of various prokaryotic and eukaryotic pathogens, and is essential for their physiology and virulence [27], [28]. To assess conservation of the subspecies-specific regions, a panel of 102 geographically and phenotypically diverse strains of *C. fetus* subspecies was screened for the presence or absence of *mat1* and *glf*. Of 62 subsp. *venerealis* isolates (all type A), 58 (93.5%) were positive for *mat1* and all were negative for *glf*. In contrast, only 16 (40%) of 40 subsp. *fetus* strains harbor *mat1* but 25 (62.5%) were positive for *glf*. The 16 subsp. *fetus* strains positive for *mat1* were all type B, whereas 24 of the 25 *glf* positive strains were type A **(Table S3, Table S6 in File S5)**. The single exception, *C. fetus* subsp. *fetus* isolate F9, which was positive for both *mat1* and *glf,* belongs to the rare group of type AB strains.

Our previous application of RDA (representational difference analysis) to *C. fetus* revealed that another LPS-biosynthesis gene (*wcbK*) encoding a putative GDP-mannose 4,6-dehydratase was exclusively present in *C. fetus* subsp. *fetus* strains [17]. In strain ATCC 27374 (type B), *wcbK* is flanked 3′ by *wbbC*, encoding a putative glycosyltransferase, and 5′ by a *sap* gene (data not shown). This region corresponds to FGI I in strain 82-40, which lacks *wcbK*. WcbK catalyzes the first step in the biosynthesis of GDP-D-rhamnose and GDP-L-fucose, and is involved in capsular polysaccharide or LPS-biosynthesis in bacteria such as *Helicobacter pylori* [29] and *C. jejuni* [30]. A PCR screen of the *C. fetus* panel confirmed that *wcbK* was not present in any of the *C. fetus* subsp. *venerealis* isolates but was exclusively detected in the 16 *C. fetus* subsp. *fetus* isolates, which were also positive for *mat1*. All of the *wcbk*+ *mat1*+ strains were type B. Thus, *C. fetus* subsp. *fetus* either carried *glf* alone in type A strains or *mat1* in combination with *wcbK* in type B strains. *C. fetus* subsp. *venerealis* (type A) only carries *mat1*. *C. fetus* subsp. *fetus* strain F9 scored positively for *mat1, wcbK* and *glf*.

Another phylotype of *C. fetus* is represented by reptile *C. fetus* strains, which are type A, and may represent the ancestral *C. fetus* type [4], [31]. We screened four reptile isolates, which were all positive for *mat1* but lacked *glf, wcbK*, *virD4* and *fic1-4* **(Table S4 in File S5)**.

Finally, another enzyme of the LPS-biosynthetic pathway UDP-glucose 4-epimerase (GalE, EC 5.1.3.2) catalyzes the reversible conversion of UDP-glucose to UDP-galactose and is known to contribute to *C. jejuni* virulence [32]. Southern-blot and PCR screens of our collection showed that all 102 *C. fetus* isolates studied carried *galE*.

### *wcbK* is Involved in LPS-biosynthesis and Accordingly should have an Impact on Acid Resistance and Serum Sensitivity in *C. fetus* subsp. *Fetus* Type B Strains

**Figure 5:**
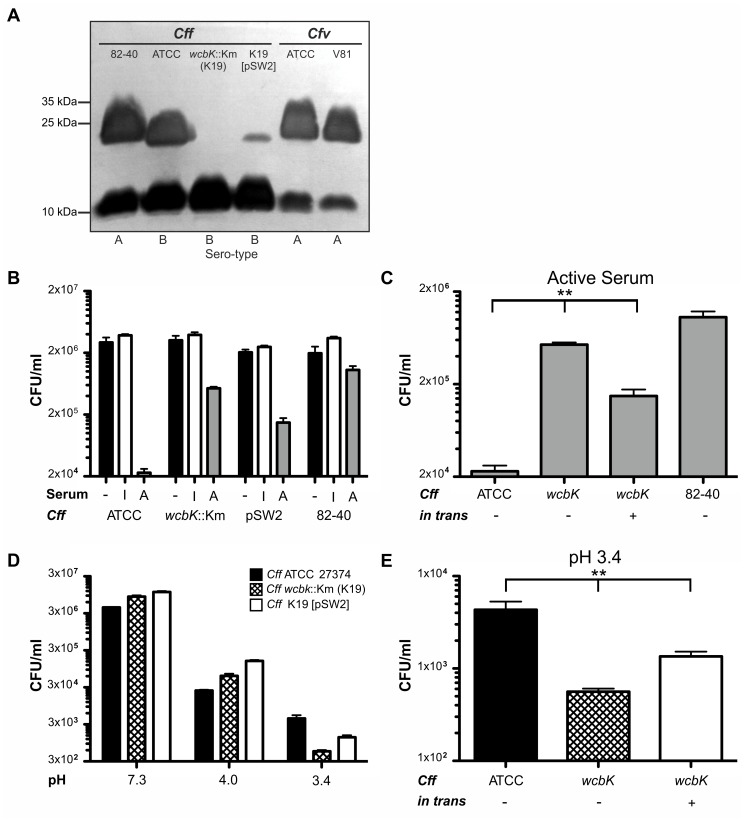
WcbK is important for LPS-biosynthesis, attenuates survival in blood, and promotes acid resistance. (**A**) SDS-PAGE pattern of purified LPS after silver staining. Samples were isolated from *C. fetus.* subsp. *fetus* (*Cff*) 82-40 (lane 1), *Cff* ATCC 27374 (type B) (lane 2), *wcbK* mutant K19 (*wcbK*::Km) (lane 3) and K19 [pSW2] (*wcbK in trans*) (lane 4); *C. fetus* subsp. *venerealis* (*Cfv*) ATCC 19438 (lane 5) and *Cfv* 84-112 (lane 6). (**B**) *Cff* serum resistance assays. Strains were incubated either with EMEM (-), heat-inactivated (I) or active (A) human serum and colony forming units (CFU) were counted. Results shown are for *Cff* ATCC 27374, K19 and K19 [pWS2]. *Cff* 82-40 served as a type A comparator. (**C**) Same as in (B) but for better visualization, CFU/ml obtained after treatment with active serum are displayed separately. **p<0.002 (**D**) Acid resistance assays. *Cff* were incubated in PBS pH range 7.3 to 3.4, plated and CFU determined. Survival after exposure to different pH of the wild type, K19 and K19 [pSW2] was compared. (**E**) For better visualization, CFU/ml for the three strains after treatment with pH 3.4 were plotted separately. **p<0.003.

Type A strains are resistant to complement-mediated killing since C3b binding to the bacterial cell surface is inhibited by the presence of the S-layer [33], [34]. It is not known why type B strains are sensitive to non-immune serum [12], despite the presence of the surface array protein. We hypothesized that *wcbK* might be linked to the susceptibility of type B strains by generating O-specific side chains where the C3b binding site is not covered by the S-layer. To test this, we first screened *C. fetus* subsp. *fetus* type A and B strains with known serum resistance phenotypes for *wcbK* and *glf* **(Table S5 in File S5)**. As hypothesized, *wcbK* was exclusively found in type B strains and correlated with serum susceptibility, whereas *glf* only was present in type A strains and correlated with serum resistance. Next we generated a non-polar *wcbK* mutant (K19) of *C. fetus* subsp. *fetus* ATCC 27374 (type B) that was deficient in LPS-production (Figure 5A). In *Vibrio cholerae* mutant strains it has been shown that providing genes in trans only partially restored LPS-production compared to wild type levels [35]. In our experiments, providing *wcbK* in trans also partially complemented LPS-production. Due to antibiotic selection throughout the experiment we can exclude the loss of the complementation vector. We next compared serum-susceptibility of mutant and wild type strains **(Figure 5BC)**. As expected, *C. fetus* subsp. *fetus* ATCC 27374 did not survive serum treatment (log_10_ kill 2.23±0.06) whereas the isogenic *wcbK* mutant strain K19 had markedly increased serum-resistance (log_10_ kill 0.86±0.05). The phenotype was partially complemented (log_10_ kill 1.23±0.10) by providing *wcbK in trans*. The serum resistant strain 82-40 (type A) was used as a control (log_10_ kill 0.27±0.01).

Type A and type B *C. fetus* strains differ in the carbohydrate composition of their LPS [12], [26]. The O-antigen of type A strain has a higher molecular weight (Figure 5A) than that of type B strains. *C. fetus* strains 84-112, 82-40 and ATCC 27374 are similar in their resistance to acid (Figure 5 and results not shown). In *H. pylori* GDP-mannose 4,6-dehydratase (encoded by *wbcJ*) is important for the expression of O-antigen and for the bacterium to survive the acidic milieu of the stomach [29]. We hypothesized that the loss of LPS in the *wcbK* deficient *C. fetus* strain might result in increased acid sensitivity. Indeed, when incubated at low pH the wild type strain (ATCC 27374) survived significantly better than the *wcbK* mutant; this acid-sensitive phenotype was partially complemented by providing *wcbK in trans* (Figure 5CD).

In summary, *wcbK* is important for LPS-biosynthesis and SapB binding. Activity of this enzyme attenuates survival of the pathogen in blood, and also can provide effective protection from stomach acid en route to colonization of the intestinal niche.

## Discussion

ε-Proteobacteria including *Campylobacter* and its close relative *Helicobacter* show evidence of genome reduction indicated by small genome size (≈1.5 to 2.5 Mbp) and the nearly complete absence of non-coding DNA. These features are typical for adaptation to a specific colonization niche and both species display strong host preference (“tropism”) [36], [37]. Among *Campylobacters*, *C. fetus* subspecies are an exceptional model system to study the molecular basis of pathogen-host adaptation since, despite a highly clonal structure, they display strikingly dissimilar host preferences and tissue tropism. To investigate the genetic basis underlying the distinct pathogenicity of *C. fetus* subspecies, we performed whole genome comparisons and transcriptome analyses of *C. fetus* subspecies, focusing on identifying differences that contribute to host and tissue tropism. We propose that the additional genome content of *C. fetus* subsp. *venerealis* was horizontally acquired **(Table S1 in File S5)**. The observation that genes shared between the subspecies are nearly 100% identical on the nucleotide level supports the hypothesis that HGT and not mutation or genetic drift is the predominant factor in the evolution of *C. fetus*.

To gain insights to the genetic plasticity of *C. fetus* genomes, and particularly whether the identified variation regions are conserved we compared the VGI – IV of *C. fetus* subsp. *venerealis* 84-112 to the draft genome sequences of *C. fetus* subsp. *venerealis* NCTC 10354 (ATCC 19438) [38], *C. fetus* subsp. *venerealis* Azul-94 [39] and *C. fetus* subsp. *venerealis* biovar Intermedius INTA 99/541 [40]. We identified homologous sequences in all three strains with over 90% homology on the nucleotide level. These results indicate that the GIs are at least partially present in other *venerealis* strains. However, given that many of the remaining contig boundaries are located in the variable regions, to be able to perform more detailed analysis the draft genomes will need to be closed and the sequences verified.

We focus in the current study on the description of genomic regions and genes unique to each subspecies. Genome comparisons of *C. fetus* subspecies reported previously using the draft sequences of *C. fetus* subsp. *venerealis* strains [39], [41] focused mainly on the description of shared putative virulence factors or the identification of putative targets for diagnostics. Many of the genes putatively involved in adherence, invasion, motility, secretion and toxin production identified by Ali *et al*. [41] and Moolhuijzen *et al*. [39] were also present in strain 84-112 (File S1). Homologs to the antibiotic resistance gene cluster identified within a homologous genomic island in *C. fetus* subsp. *fetus* IMD 523-06 [42] were not present in *C. fetus* subsp. *venerealis* ATCC and 84-112.

Metabolic differences between *C. fetus* subspecies such as glycine tolerance, H2S production and selenite reduction have traditionally been used to discriminate the subspecies and are therefore intriguing features linked to niche adaptation. Nonetheless, metabolic modeling of the two genomes revealed no apparent subspecies differences, except a possible difference in thiamin (vitamin B1) biosynthesis. The overall metabolic capacity seems to be similar in both subspecies, consistent with our model that the described horizontally acquired genetic elements account for the different biology of *C. fetus* subspecies. However, it is important to note that subtle genetic differences, like point mutations, can inactivate genes or disrupt metabolic pathways. Therefore, nutrient utilization by the *C. fetus* subspecies remains an important priority for detailed study.

The extra-chromosomal element ICE_84-112 was identified. ICEs are plasmid-like self-transmissible mobile genetic elements, dependent on phages or transposons for inserting and excising from chromosomes, but carry their own transfer genes (*tra*-genes) for lateral transmission to other host cells. Notably the full repertoire of plasmid replication genes is typically absent. Some ICE replicate autonomously if they adopt a rolling-circle-like mechanism mediated by replication- or single-strand DNA transfer initiation factors [20], [21]. In *Bacillus subtilis* helicase PcrA associates with ICE*Bs1* during replication [20]. Candidate PcrA orthologs are present in both *C. fetus* subspecies (84-112 CDS peg.56 & peg.1280 (**File S1**) and 82-40 CDS peg.690 & peg.934 (**File S2**)). The surveyed *fic3* and *fic4* genes suggest that the distribution of ICE_84-112 is quite narrow. In that case important virulence-associated characteristics are unlikely to be carried by the element, but it may be a vehicle of interspecies gene exchange.

**Figure 6:**
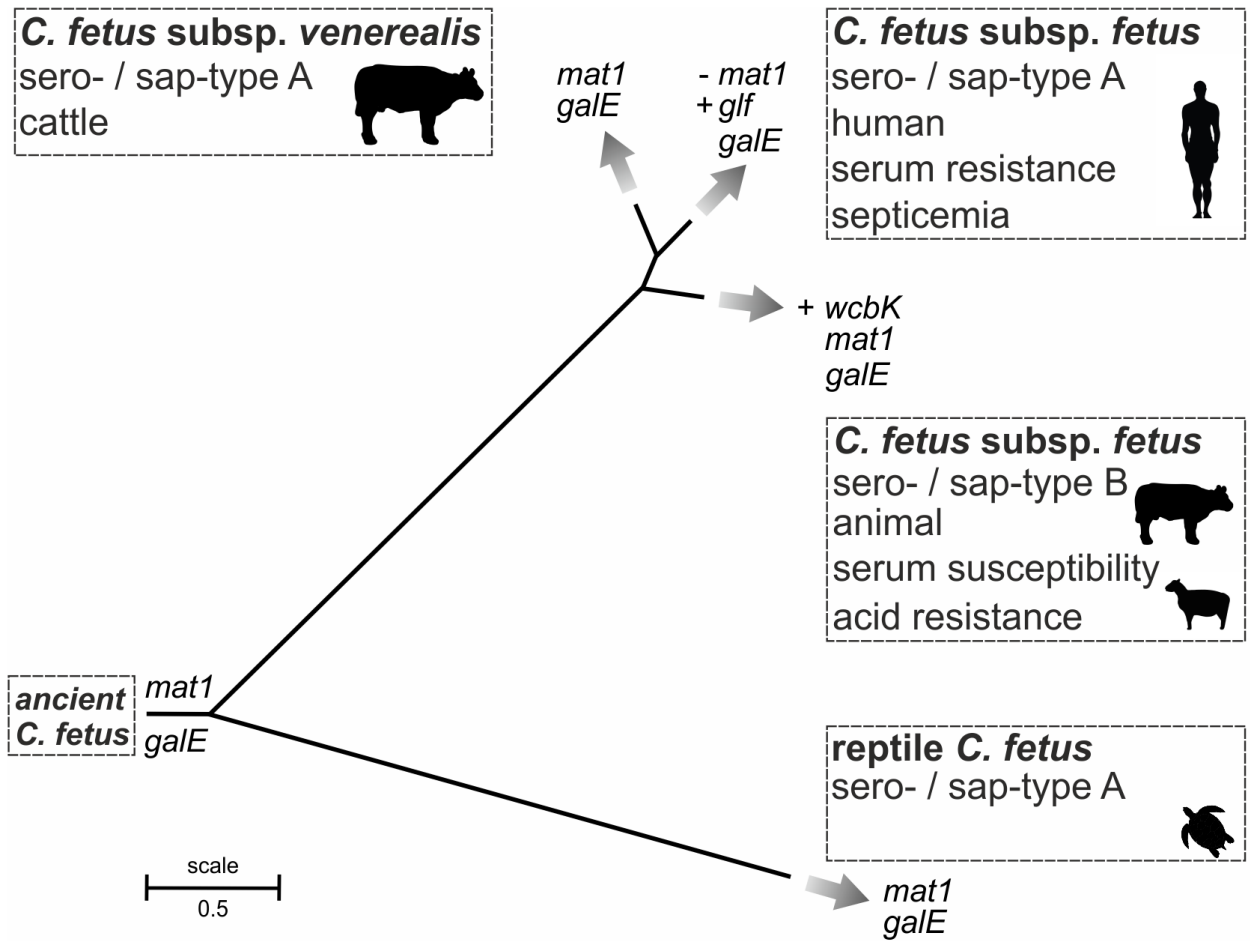
Phylogeny, niche specificity and virulence of *C. fetus* subspecies. MLST tree showing the phylogeny of *C. fetus,* with original scale as reported [4]. Reptile *C. fetus* represent a distinct clade harboring *mat1* and *galE*. Diversification of *C. fetus* subsp. *fetus* (*Cff*) type B happened prior to the diversification of *Cff* type A and *C. fetus* subsp. *venerealis* (*Cfv*) type A strains. *Cff* type B strains harbor *galE*, *mat1* and *wcbK*. The latter gene provides protection from acid, and this genotype is associated with animal hosts. *Cfv* type A represents the bovine clone harboring *mat1* and *galE* which is also prone to HGT. *Cff* type A have lost *mat1* but acquired *glf* correlating with serum resistance in *Cff*.

*C. fetus* subsp. *fetus* 82-40 mostly lacks phage- and plasmid-related genes and this might be due to the presence of an active CRISPR cluster, protecting from invasion of foreign DNA. Although there are six core *cas*-genes, *cas1* may be of central importance in the acquisition of new spacers (for review see [43]). In contrast to *C. fetus* subsp. *venerealis* 84-112, we identified two CRISPR-arrays in strain 82-40. Since Cff_CRISPR_2 showed prototypical architecture, i.e., *cas*-genes and an AT-rich leader sequence followed by the DRs and the spacers, this CRISPR array may be functional. The presence of *cas*-genes in *C. fetus* subsp. *fetus* highlights another important subspecies difference. The occurrence of CRISPRs is linked to natural competence of bacteria [44]. That *C. fetus* subsp. *fetus* type B strains more frequently harbor putative functional CRISPRs than type A strains might have stabilized the type B phylotype and may explain why the type A clade later diverged [4] (Figure 6). All of the *C. fetus* strains that we and others have thus far tested are not naturally competent (unpublished data, [45], [46]) thus a possible connection between the presence of CRISPRs and natural competence of *C. fetus* subspecies remains unresolved.

The most important genetic differences between the subspecies are cell surface structures including the S-layer and LPS. The distribution of these genes across a panel of diverse *C. fetus* isolates indicates linkage to particular pathotypes. The distinct distribution patterns detected for *wcbK*, *mat1*, and *glf* among type A and B strains support the following model **(** **Figure 6** **)**. *wcbK and glf* are subsp. *fetus*-specific genes that have been acquired more recently than *mat1* and *galE, which* represent “ancient” constituents of the *C. fetus* genome. These loci are similar in reptile *C. fetus* and *C. fetus* subsp. *venerealis* but MLST reveals that variation has emerged and that type B strains separated from type A prior to the division of *C. fetus* subsp. *fetus* and *C. fetus* subsp. *venerealis* [4]. We showed that type B strains maintained *mat1* and *galE* but diversification of phylotypes led to acquisition of *wcbK* by *C. fetus* subsp. *fetus* type B. *C. fetus* subsp. *venerealis* also maintained *mat1* and *galE*, but type A *C. fetus* subsp. *fetus,* the invasive pathotype often found in human infections, have lost *mat1* and acquired *glf*. Extended analysis of *C. fetus* evolution will require analysis of more geographically and phenotypically diverse isolates. Moreover, analysis of the newly proposed subspecies/biovar intermedius [7] may provide a missing link in the subspecies divergence.

Little is known how *C. fetus* interacts with the host immunity, but LPS and the S-layer are important for TLR4-mediated recognition [47], [48]. The S-layer producing *C. rectus* induces TLR4 expression in the mouse placenta [49]. To avoid dysregulated inflammatory responses to LPS, the intestinal epithelium as well as placental tissue normally express no or low levels of TLR4 [48], [50], [51]. Low density of TLR4 may allow *C. fetus* to overcome the hosts’ immune response and subsequently invade the host cells. Type A and type B *C. fetus* strains are different in their LPS composition and S-layer proteins [12], [26]. The activity of WcbK and the putative functions of *mat1* and *glf* are linked to the S-layer. *C. fetus* subsp, *venerealis* strains (*wcbk*−/*glf*−/*mat1*+) and *C. fetus* subsp. *fetus* type A strains (*wcbK*−/*glf*+/*mat1*-) are serum resistant, whereas *C. fetus* subsp. *fetus* type B strains (*wcbK*+/*glf*−/*mat1*+) are serum sensitive. We showed that *wcbK* is essential for LPS-biosynthesis in *C. fetus* subsp. *fetus* type B strains and that loss of *wcbK* leads to increased serum resistance. This data indicates that WcbK generated side chains are important for serum sensitivity. We propose that similar to *wcbK*, the products of *mat1* and *glf* of *C. fetus* might be involved in LPS-biosynthesis by generating different O-antigen side chains, potentially influencing complement and antibody binding, acid resistance and TLR-4 recognition.

The bacterial transcriptome provides an additional reference to study genome composition as well as regulation of virulence. In the initial profile of *C. fetus* gene transcription, the characteristic ε-proteobacterial promoter signature was identified. We confirmed that the promoter region is 100% conserved between the subspecies, and that one *sap* gene is predominantly transcribed under laboratory conditions. This finding is intriguing since recombination and therefore exchange of *sap*-homologs occurs frequently in this region to enable phase variation of the pathogen [11]. It has been proposed that the *sap*-region belongs to the ancestral part of the *C. fetus* core genome and not a PAI [52]. That the region is shared between both subspecies confirms ancient presence of a horizontally acquired element. Based on the significance of the S-layer for immune evasion [11], [15], [16], the genome insertion can be considered as a classical PAI. To date, animal models of *C. fetus* infection are not readily available. Future analyses at the transcriptiome level should investigate *C. fetus* under *in vitro* conditions resembling their colonization niche or route of infection.

Whole-genome comparisons of related pathogens of distinct characteristics, such as those described in the presented work, lay the foundation for additional mutational, functional, and animal studies that will ultimately help elucidate the mechanisms underlying the emergence of new pathogens. This study broadens knowledge of the genomic basis of *C. fetus* pathogenesis and host specificity. The most interesting differences in the genetic repertoire of the subspecies relate to cell surface structures including the S-layer and LPS and distribution of these genes is associated with certain pathotypes. This emphasizes the importance of surface-exposed structures to *C. fetus* pathogenicity and demonstrates how evolutionary forces optimize the fitness and host adaptation of these pathogens. The presence of genes like *glf* is particularly interesting as the gene product is a promising drug target, as proposed for *Leishmania* [53], and relevant since *glf* is connected to type A strains, which are more often isolated from human blood. In any event, *wcbK* and *glf* are excellent candidates applicable for reliable subspecies differentiation.

## Experimental Procedures

### Bacterial Strains

*Campylobacter* and *E. coli* strains were grown as described [45]. Antibiotic selection applied concentrations of 100 µg ml^−1^ ampicillin, 75 µg ml^−1^ nalidixic acid, or kanamycin and chloramphenicol at 25 µg ml^−1^. Bacterial strains are listed in **Table S6** and **Table S7 in File S5**. Only *C. fetus* strains typed definitively to the subspecies level were tested in PCR screens (n = 102). Subspecies were identified biochemically as described [17].

### Gene Detection

Oligonucleotides are listed in **Table S8 in File S5**. PCR amplification for surveying gene prevalence used chromosomal DNA and the following primer pairs 1/2 for *wcbK*, 3/4 for *glf* and 5/6 for *mat1*. The *sap*-type was determined with primers 7/8 and 9/10, as described [54]. Southern blots were hybridized with radiolabeled DNA probes as described [17]. Probes for *galE* and *cas1* were generated with primer pair 11/12 and 13/14 from chromosomal DNA of *C. fetus* subsp. *fetus* ATCC 27374, respectively. The same primers were used for PCR-screening for *galE* and *cas1*. *fic3* and *fic4* were amplified with primer pairs 15/16 and 17/18, respectively.

### Genome Sequencing, Assembly and Annotation

A standard whole genome shot-gun and a 3-kb paired-end library were generated according to the manufacturer’s recommendations (Roche Diagnostics, Vienna, Austria) using 5 µg chromosomal DNA. For each library, high-throughput pyrosequencing was performed on a Genome Sequencer FLX system (Roche) producing 145 Mb and 62.2 Mb sequence data, respectively. Read assembly applied the Newbler assembly software, version 2.6 (Roche) and resulted in 89 contigs and 11 scaffolds. One scaffold represented the circular extra-chromosomal element and the remaining 10 were grouped into 3 super-scaffolds (SSc) using the information from the 3 kbp mate-pair library and the contig-graph generated by the Newbler assembler. Additionally, PCR and Sanger sequencing was used to determine the orientation and order of contigs and the SSc. Gaps in the extra-chromosomal element and the chromosome were closed *in silico* with a custom R script [55] and with PCR. Homopolymer uncertainties from the 454-reads were corrected through mapping of the Illumina reads derived from the *C. fetus* subsp. *venerealis* 84-112 RNA to the draft sequence using CLC Genomics Workbench 5.5 (CLC Bio; Arhus, Denmark). The resulting consensus sequences and *C. fetus* subsp. *fetus* strain 82-40 were annotated and compared with Rapid Annotations using Subsystem Technology version 4.0 (RAST) [56]. Annotation tables for each strain and the extra-chromosomal element are presented in **Files S1–S3**.

### Differential RNA-sequencing

Library preparation for dRNA-seq was performed as reported [23]. In brief, RNA was isolated from bacterial cells grown on CBA plates for 24 h. To construct differential cDNA library pairs, aliquots of extracted RNA from each strain was treated with Terminator-5′-phosphate-dependent exonuclease (TEX; Epicentre) to deplete processed RNAs (denoted TEX+) in addition to untreated RNA (denoted TEX-). Construction of cDNA libraries was performed by *vertis* Biotechnology AG (Munich, Germany). Libraries were sequenced using cluster amplification with the TruSeq PE Cluster Kit v.5 on a cluster station. Each library was sequenced on a single HiSeq 2000 lane using TruSeq SBS 36 Cycle Kits v.5 (Illumina, San Diego, CA) and a 91 bp single-end protocol. Sequencing image files were processed with the Sequencing Control Software (SCS) Real Time Analysis (RTA) v2.6 and CASAVA v.1.7 (Illumina). Reads were mapped to the reference genomes using the CLC Genomics workbench (CLC Bio) with default settings. Information on transcriptional start site (TSS) and promoter annotation can be found in the supplement.

### Lipopolysaccharide Analysis

*C. fetus* strains were grown for 24 h and resuspended in buffer (10% glycerine, 20% SDS, 5% β-mercaptoethanol, 62.5 mM Tris-HCl pH 6.8, bromophenol blue) for lysis at 100°C for 10 min. Proteinase K solution was added to 6 µg/µl and samples were incubated overnight at 55°C. LPS-preparations were electrophoretically on 15% polyacrylamide gels (running buffer: 86 mM glycine, 3,5 mM SDS and 25 mM Tris pH 8). Gels were fixed overnight (25% isopropanol, 7% acetic acid) under gentle shaking. LPS was oxidized with 100 ml fixative containing 4 mmol NaIO_4_ for 10 min. After three washing steps with H_2_O for 30 min each, the gels were stained (19 mM NaOH, 1.35% NH_3_, 20 mM AgNO_3_) for 10 min, then washed three times with H_2_O and immersed in developer (240 mM Na_2_CO_3_, preheated to 60°C, before addition of 30 µl 40% formaldehyde). The reaction was stopped with 50 mM EDTA (pH 8) for 1 h.

### Serum and Acid Resistance Testing

Susceptibility of *C. fetus* strains to human serum was assessed as described [57]. All tests were performed in triplicate. Briefly, *C. fetus* was streaked on CBA plates 24 h prior to the assay and cell count was adjusted to 1×10^7^ bacteria/ml, based on optical density in EMEM medium. The actual cell count was determined by plating serial dilutions. Heat-inactivated- (56°C for 30 min), or active- (thawed on ice) pooled human serum was added to the bacteria to a 10% final concentration and incubated for 1 h at 37°C. Surviving cells were counted on CBA plates after 48 h growth. For the acid resistance assays, *C. fetus* cells were harvested as described above, centrifuged, resuspended in PBS with different pH values and incubated at 37°C for 30 min. Cells were washed in PBS (pH 7.3) before the number of surviving bacteria was determined by plating serial dilutions.

### Nucleotide Sequence Accession Numbers

The genome sequence of *C. fetus* subsp. *venerealis* 84-112 including the ICE element (ICE_84-112) has been deposited in EMBL Nucleotide Archive under accession numbers (HG004426 and HG004427). The genome of *C. fetus* subsp. *fetus* 82-40 used for comparative analyses has the GenBank accession number CP000487.1. dRNAseq data can be accessed via the EMBL-EBI short read archive under the accession number ERP002581.

## Supporting Information

Figure S1 **Comparative maps of CRISPR-related genomic islands. (A)** *C. fetus* subsp. *venerealis* 84-112 VGI IV harbors the direct repeats with spacers (CRISPR) but lacks CRISPR-associated (*cas)*-genes. Prophage-related genes (putative prophage IV) were identified (orange) adjacent to a region identical to *C. fetus* subsp. *fetus* 82-40 Downstream of these regions the core-genome continues with a chromosomal rearrangement between the two subspecies on the 3-prime end (striped boxes). A sequence region shared between the subspecies was identified (blue box). **(B)** *C. fetus* subsp. *fetus* 82-40 FGI I carries two regions of direct repeats and spacers. *cas*-genes precede the second CRISPR-array resulting in a putatively functional CRISPR-system. One region with a prophage-like structure (orange) was identified.
(TIF)

Figure S2 **Physical map of the extra-chromosomal element ICE_84-112.** Shown is the GC-content (circle 1), GC-skew (circle 2) and open reading frames (circle 3). The *tra*-region (red) comprises genes putatively involved in conjugative transfer of the ICE. The *vir*-region (orange) shows putative T4SS genes with homology to the chromosomal PAI on VGI I. Genes possibly involved in autonomous replication of the ICE are named individually and labeled (green and red). Genes of predicted plasmid origin (green); phage genes and transposons (blue); putative effector proteins or toxin-antitoxin system (yellow); hypothetical proteins (grey).
(TIF)

Figure S3 **Schematic representation of the apparent T4SS identified in** ***C. fetus*** **subsp.** ***venerealis*** **84-112. (A, B, C)** Represent loci with homology to *virB/virD4*-genes. **(A)** The PAI T4SS is functional in virulence and conjugative DNA transfer [1], [2]. **(B)** ICE_*vir* displays a similar gene organization to VGI I but protein homologies are not strikingly high. *virD4* is truncated compared to the functional PAI homologue. **(C)** A partial set of *vir*-genes. **(D)** ICE_*trb*/*tra* genes share homology to plasmid RP4 and are putatively involved in the conjugative transfer of ICE_84-112. Homologous genes (*vir, tra*) are indicated by color.
(TIF)

Figure S4 **Comparative map of** ***C. fetus*** **subspecies variation regions VSDR and FSDR. (A)** *C. fetus* subsp. *venerealis* 84-112 VSDR and **(B)** *C. fetus* subsp. *fetus* 82-40 FSDR. MAUVE was used to compare the regions to visualize rearrangements and insertions. Regions free of rearrangements are indicated by colored colinear blocks. White regions within these blocks symbolize insertions or non-homologous regions. Important open reading frames are colored and/or labeled accordingly. Genes unique to the subspecies, *mat1* and *glf*, are highlighted in pink.
(TIF)

Figure S5 **Venn diagram of annotated TSS. (A)** *C. fetus* subsp. *venerealis* 84-112 and **(B)** *C. fetus* subsp. *fetus* 82-40. TSS were categorized according to the genomic context into five classes: primary (TSS having the most cDNAs within ≈500 bp upstream of annotated mRNA start codons), secondary (TSS associated with the same gene but with fewer cDNAs), internal (TSS within an annotated gene on the same strand), antisense (TSS situated inside or within ≈100 bp of the coding region of a gene encoded on the opposite strand), or orphan (TSS without annotated genes in proximity) [3]. Numbers in parentheses indicate the TSS, which associate with only one *orf*.
(TIF)

File S1 (XLSX)

File S2 (XLSX)

File S3 (XLSX)

File S4 (XLSX)

File S5 (DOC)
