# Supplementary figures and images for: Comparative Genome Analysis of Campylobacter fetus Subspecies Revealed Horizontally Acquired Genetic Elements Important for Virulence and Niche Specificity
Source: PLoS One. 2014 Jan 9;9(1):e85491. doi: 10.1371/journal.pone.0085491 (PMC3887049; doi:10.1371/journal.pone.0085491)

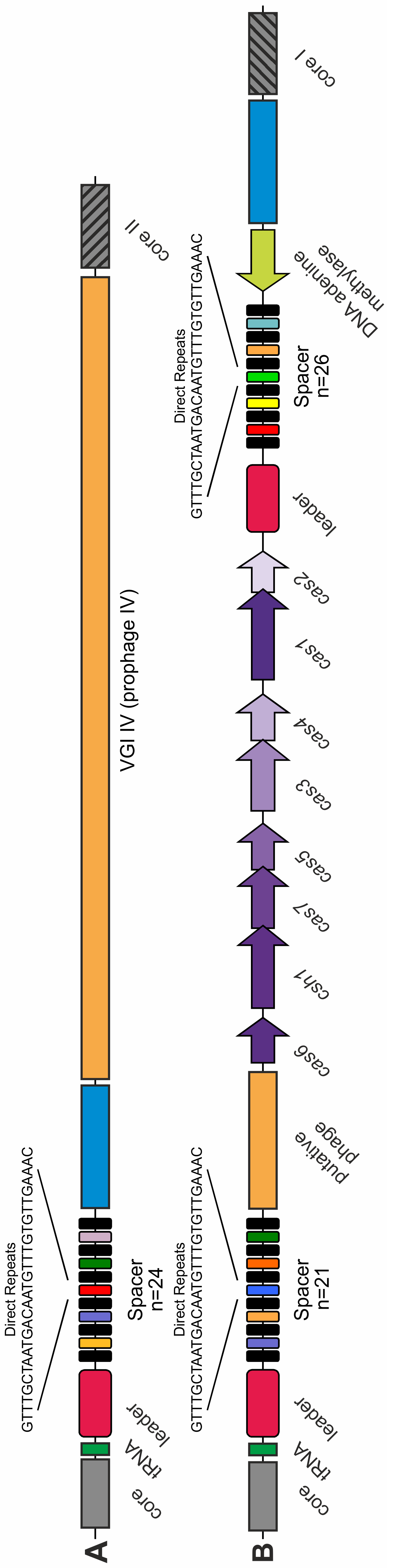

Supplement: Figure S1 — Comparative maps of CRISPR-related genomic islands. (A) C. fetus subsp. venerealis 84-112 VGI IV harbors the direct repeats with spacers (CRISPR) but lacks CRISPR-associated (cas)-genes. Prophage-related genes (putative prophage IV) were identified (orange) adjacent to a region identical to C. fetus subsp. fetus 82-40 Downstream of these regions the core-genome continues with a chromosomal rearrangement between the two subspecies on the 3-prime end (striped boxes). A sequence region shared between the subspecies was identified (blue box). (B) C. fetus subsp. fetus 82-40 FGI I carries two regions of direct repeats and spacers. cas-genes precede the second CRISPR-array resulting in a putatively functional CRISPR-system. One region with a prophage-like structure (orange) was identified. (TIF) [file pone.0085491.s001.tif]

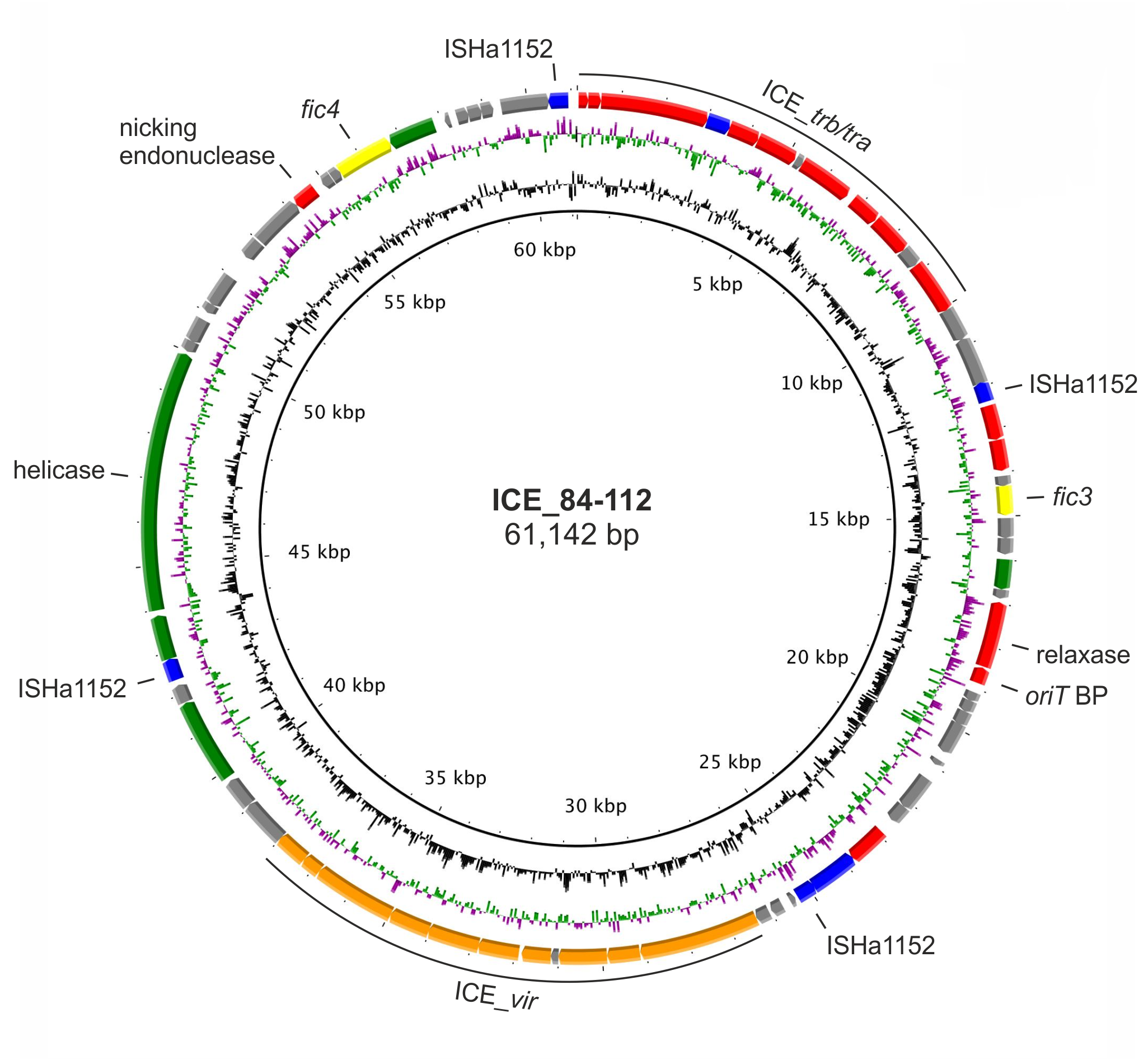

Supplement: Figure S2 — Physical map of the extra-chromosomal element ICE_84-112. Shown is the GC-content (circle 1), GC-skew (circle 2) and open reading frames (circle 3). The tra-region (red) comprises genes putatively involved in conjugative transfer of the ICE. The vir-region (orange) shows putative T4SS genes with homology to the chromosomal PAI on VGI I. Genes possibly involved in autonomous replication of the ICE are named individually and labeled (green and red). Genes of predicted plasmid origin (green); phage genes and transposons (blue); putative effector proteins or toxin-antitoxin system (yellow); hypothetical proteins (grey). (TIF) [file pone.0085491.s002.tif]

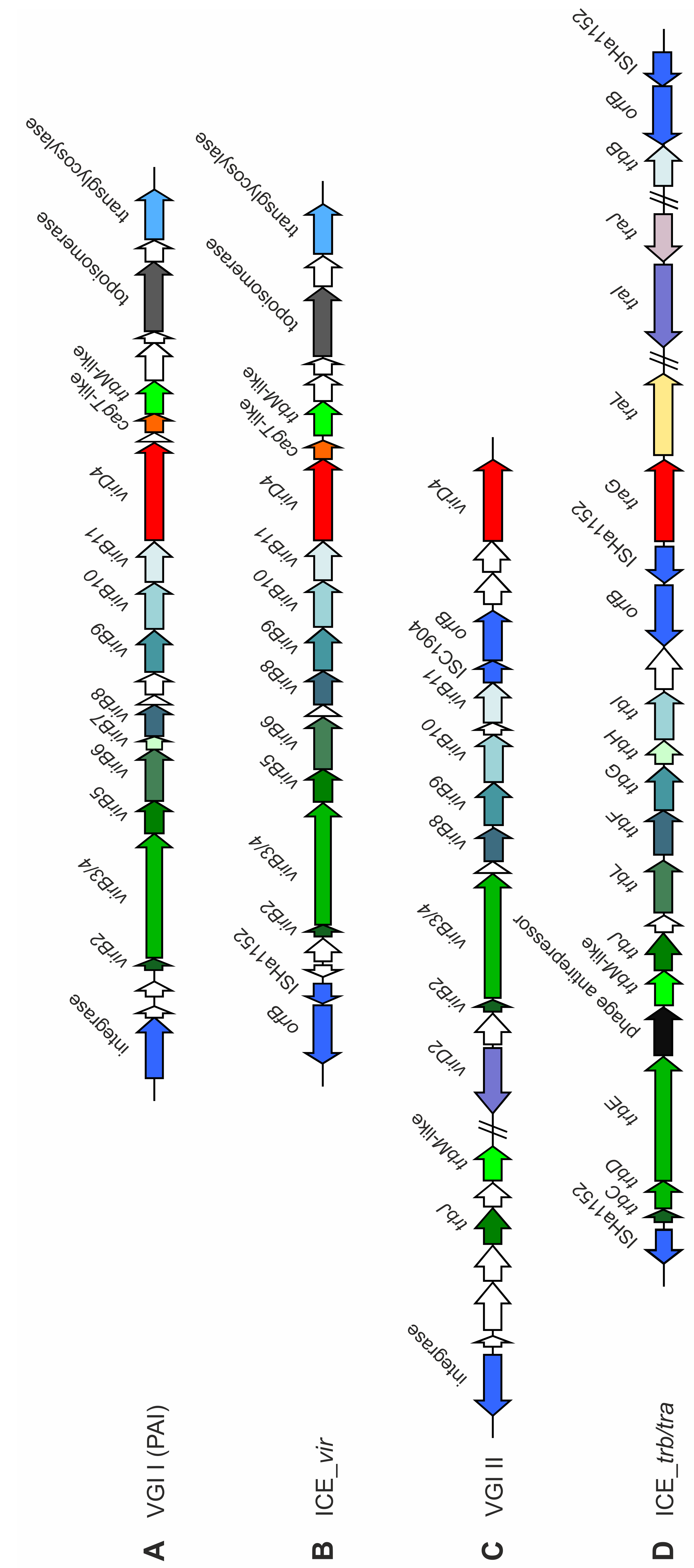

Supplement: Figure S3 — Schematic representation of the apparent T4SS identified in C. fetus subsp. venerealis 84-112. (A, B, C) Represent loci with homology to virB/virD4-genes. (A) The PAI T4SS is functional in virulence and conjugative DNA transfer [1], [2]. (B) ICE_vir displays a similar gene organization to VGI I but protein homologies are not strikingly high. virD4 is truncated compared to the functional PAI homologue. (C) A partial set of vir-genes. (D) ICE_trb/tra genes share homology to plasmid RP4 and are putatively involved in the conjugative transfer of ICE_84-112. Homologous genes (vir, tra) are indicated by color. (TIF) [file pone.0085491.s003.tif]

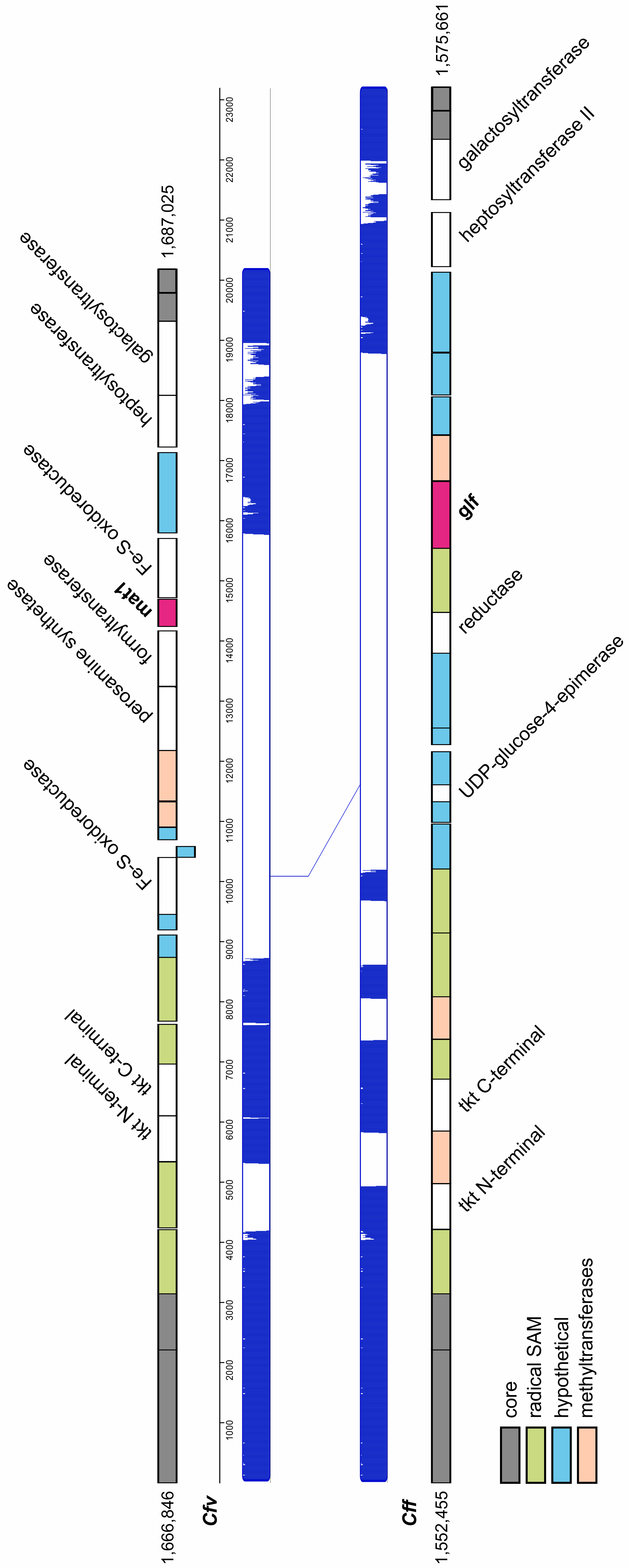

Supplement: Figure S4 — Comparative map of C. fetus subspecies variation regions VSDR and FSDR. (A) C. fetus subsp. venerealis 84-112 VSDR and (B) C. fetus subsp. fetus 82-40 FSDR. MAUVE was used to compare the regions to visualize rearrangements and insertions. Regions free of rearrangements are indicated by colored colinear blocks. White regions within these blocks symbolize insertions or non-homologous regions. Important open reading frames are colored and/or labeled accordingly. Genes unique to the subspecies, mat1 and glf, are highlighted in pink. (TIF) [file pone.0085491.s004.tif]

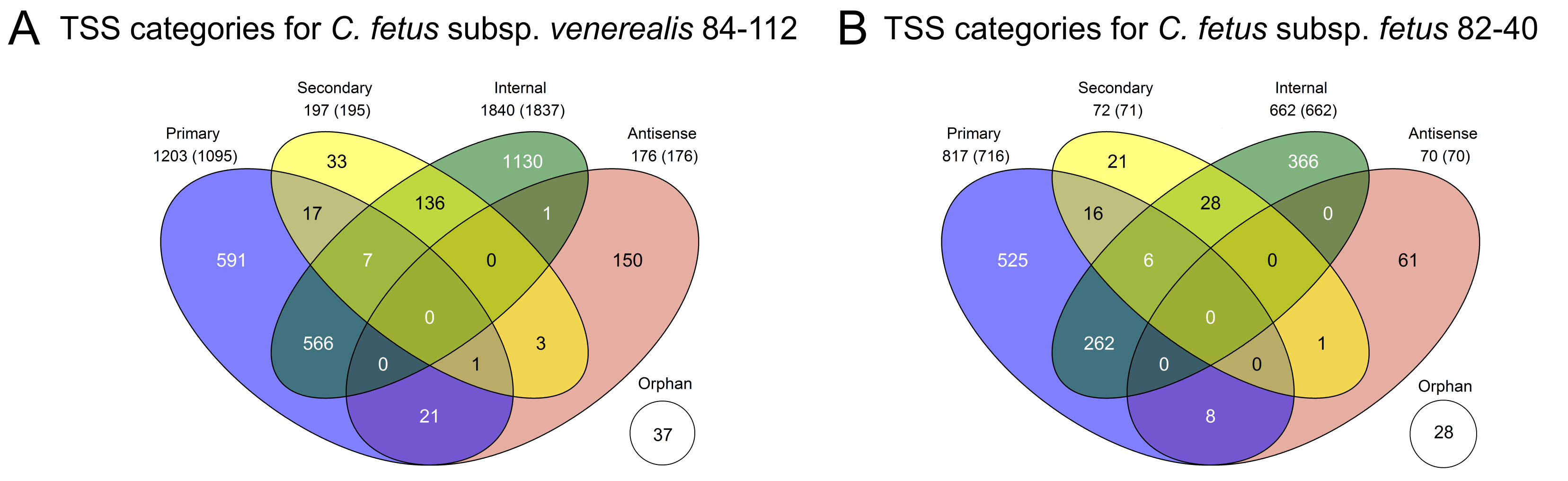

Supplement: Figure S5 — Venn diagram of annotated TSS. (A) C. fetus subsp. venerealis 84-112 and (B) C. fetus subsp. fetus 82-40. TSS were categorized according to the genomic context into five classes: primary (TSS having the most cDNAs within ≈500 bp upstream of annotated mRNA start codons), secondary (TSS associated with the same gene but with fewer cDNAs), internal (TSS within an annotated gene on the same strand), antisense (TSS situated inside or within ≈100 bp of the coding region of a gene encoded on the opposite strand), or orphan (TSS without annotated genes in proximity) [3]. Numbers in parentheses indicate the TSS, which associate with only one orf. (TIF) [file pone.0085491.s005.tif]
