# Supplementary material for: Comparative Genome Analysis of Campylobacter fetus Subspecies Revealed Horizontally Acquired Genetic Elements Important for Virulence and Niche Specificity
Source: PLoS One. 2014 Jan 9;9(1):e85491. doi: 10.1371/journal.pone.0085491 (PMC3887049; doi:10.1371/journal.pone.0085491)
Supplement: File S5 — (DOC) [file pone.0085491.s010.doc]

**SUPPORTING INFORMATION**

**Table S1. Location and attributes of genomic islands of *C. fetus*.**

| **Designation** | | | **Position (nt)** | **tRNA** | **GC %** | **Content** | **Comment** |
| --- | --- | --- | --- | --- | --- | --- | --- |
| ***C. fetus* subsp. *venerealis* 84-112** | | | | | | |  |
| VGI I | 1,266,041 - 1,330,304 | | | Yes | 34.72 | PAI, T4SS-operon, prophage I | insertion |
| VGI II | 1,833,299 - 1,870,694 | | | Yes | 30.79 | *vir*-, plasmid related genes | insertion |
| VGI III | 529,091 - 616,674 | | | Yes | 34.41 |  |  |
| VGI IIIA-1 | 529,091 - 572,998 | | |  | 34.43 | *sap*-locus | similar to FGI I |
| VGI IIIB | 573,097 - 604,459 | | |  | 35.84 | prophage III | Insertion |
| VGI IIIA-2 | 604,695 - 616,674 | | |  | 34.43 | *sap*-locus | similar to FGI I |
| VGI IV | 718,328 - 762,382 | | | Yes | 34.30 | prophage IV | similar to FGI II |
| VSDR | 1,669,985 - 1,683,977 | | | No | 30.66 | *mat1* |  |
| ***C. fetus* subsp. *fetus* 82-40** | | | | | | |  |
| FGI I | | 433,700 - 492,574 | | Yes | 34.81 | *sap-*locus |  |
| FGI II | | 654,904 - 678,662 | | Yes | 32.64 | CRISPRs*, cas-*genes, |  |
| FSDR | | 1,555,594 - 1,572,586 | | No | 29.37 | *glf* |  |

**Table S2. Protein homologies between VirB4 and VirB11 of selected T4SS.**

|  |  | **VirB4** | | | | | | | |  |  |
| --- | --- | --- | --- | --- | --- | --- | --- | --- | --- | --- | --- |
|  |  | **1** | **2** | **3** | **4** | **5** | **6** | **7** | **8** |  |  |
| **VirB11** | **1** |  | 55.5 | 20.8 | 18.8 | 56.6 | 19.1 | 21.5 | 18.5 | **1** | **VGI I, PAI** |
|  | **2** | 63.7 |  | 20.4 | 18.9 | 65.6 | 18.8 | 21.0 | 18.2 | **2** | **ICE_*vir*** |
|  | **3** | 24.9 | 23.2 |  | 16.7 | 24.1 | 16.0 | 80.7 | 16.1 | **3** | **VGI II** |
|  | **4** | 26.8 | 25.8 | 23.6 |  | 22.1 | 73.7 | 17.4 | 44.5 | **4** | **ICE_*trb/tra*** |
|  | **5** | 63.0 | 68.8 | 26.0 | 29.8 |  | 22.5 | 24.2 | 20.5 | **5** | ***C. hominis*** |
|  | **6** | 26.3 | 28.0 | 23.6 | 72.8 | 27.1 |  | 16.1 | 42.5 | **6** | ***C. jejuni*** |
|  | **7** | 24.2 | 23.4 | 85.5 | 23.0 | 24.7 | 22.9 |  | 16.6 | **7** | ***C. rectus*** |
|  | **8** | 24.4 | 25.7 | 22.4 | 47.5 | 26.1 | 50.5 | 23.4 |  | **8** | **RP4** |
|  |  | **1** | **2** | **3** | **4** | **5** | **6** | **7** | **8** |  |  |

**Table S3. *wcbK* and *glf* correlate with the sap-type of *C. fetus*** subspecies.

|  | ***C. fetus* subsp*.*** | |  | **Genotype** | | | | **sap-type** | | | |
| --- | --- | --- | --- | --- | --- | --- | --- | --- | --- | --- | --- |
|  |  | # |  | *glf* | *wcbK* | *mat1* | *galE* |  | A | B | AB |
| a) | *Cf. venerealis* | 62 |  | 0 | 0 | 58 | 62 |  | 62 | 0 | 0 |
|  | *Cf. fetus* | 40 |  | 25 | 16 | 16 | 40 |  | 24 | 15 | 1 |
| b) | *Cf. fetus* human | 20 |  | 17 | 3 | 3 | 20 |  | 17 | 3 | 0 |
|  | *Cf. fetus* animal | 20 |  | 8 | 13 | 13 | 20 |  | 7 | 12 | 1 |

**Table S4. Gene distribution in reptile *C. fetus* isolates.**

| *C. fetus* | sap-type | *galE* | *glf* | *wcbK* | *mat1* | *virD4* | *fic1* | *fic2* | *fic3* | *fic4* |
| --- | --- | --- | --- | --- | --- | --- | --- | --- | --- | --- |
| 03-427 | A | + | - | - | + | - | - | - | - | - |
| 03-445 | A | + | - | - | + | - | - | - | - | - |
| 85-388 | A | + | - | - | + | - | - | - | - | - |
| 07-105 | A | + | - | - | + | - | - | - | - | - |

**Table S5. *wcbK* and *glf*** correlate with serum resistance and sensitivity.

| *C. fetus* | Source | *wcbK* | *glf* | *mat1* | sap-type | Serum* |
| --- | --- | --- | --- | --- | --- | --- |
| Cff 80-109 | Human blood | - | + | - | A | R |
| Cff 81-173 | Human cerebrospinal fluid | - | + | - | A | R |
| Cff 81-200 | Human feces | - | + | - | A | R |
| Cff 84-88 (1286) | Human blood | - | + | - | A | R |
| Cff 82-40 | Human blood | - | + | - | A | R |
| Cff ATCC 27374 | Sheep brain | + | - | + | B | S |
| Cff 84-91 (1366) | Human blood | + | - | + | B | S |
| Cff 84-94 (1367) | Human blood | + | - | + | B | S |
| Cff 83-88 | Human blood | + | - | + | B | S |
| Cfv 84-112 | Bovine, genital secretion | - | - | + | A | R |

Cff, *C. fetus* subsp. *fetus*; Cfv, *C*. *fetus* subsp. *venerealis*: *Serum resistance phenotype, as previously determined ; (R) resistant, (S) sensitive;

**SUPPLEMENTAL EXPERIMENTAL PROCEDURES**

**Plasmid and *C. fetus* mutant strain construction.** Plasmids and cloning intermediates are described in **Table S7** and oligonucleotides are listed in **Table S8.** A suicide vector was prepared for inactivation of *wcbK*. The IncP-type *nic* site was amplified from pRYSS1 with the primer pair 19/20 and inserted into pBluescriptIIKS(-) to generate pBlue-mob. Next, a fragment containing the *C. fetus* *gatC* promoter and the *aphA-3* kanamycin resistance cassette was excised from pRYBM5 with EcoRI and NotI and ligated into an EcoRI/NotI-digested pBlue-mob to create pBlue-mob-Km. To generate the non-polar *C. fetus* subsp. *fetus* *wcbK* deletion strain K19, the gene regions flanking *wcbK* were amplified from *C. fetus* subsp. *fetus* ATCC 27374 chromosomal DNA. A 743 bp 3-prime flanking region of *wcbK* containing 325 bp *wcbK* and 418 bp *sapB2* was amplified using the primer pair 21/22, and ligated into pBlue-mob. In the resulting plasmid the *wcbK* 5-prime flanking region, amplified from the chromosome with primer pair 23/24 was inserted. The suicide vector, pSW1, was transferred into *C. fetus* subsp. *fetus* ATCC 27374 via conjugation using *E. coli* S17-*λpir* as described . For complementation analysis, *wcbK* was amplified with the primer pair 25/26, and inserted into pRYSK12 to generate pSW2.

**Additional information for genome analysis.** Genome and regional alignments were performed with MAUVE genome alignment software 2.3.1 . Genome plots were created using the BLAST Ring Image Generator (BRIG) . Genomic islands and prophages were assessed using IslandViewer and PHAST , respectively. CRISPRs were identified using the CRISPRfinder program .

**Additional information on dRNAseq, transcriptional start site (TSS) and promoter annotation.** cDNA libraries yielded 5,548,661 (TEX+) and 12,510,083 (TEX-) reads for *C. fetus* subsp. *fetus* 82-40 and 5,222,345 (94.1%; TEX+) and 11,021,336 (88.1%; TEX-) of these could be mapped to the genome. For *C. fetus* subsp. *venerealis* 84-112, 9,491,477 (TEX+) and 12,621,193 (TEX-) reads were generated and 8,868,431 (93.4%; TEX+) and 11,309,554 (89.6%; TEX-) could be mapped to the genome. TSS identification was done computationally. Coverage values below seven were set to zero. A TSS was defined if coverage increased steeply within one nucleotide position from 0 to >17. Only those TSS that were separated at least 85 nucleotides from each other, were included in the analyses since average read length was 91 bp. This resulted in 646 TSS on the leading and 574 TSS on the lagging strand of the 82-40 genome and 1,457 TSS on the leading and 1,132 TSS on the lagging strand of genome 84-112. Promoter consensus sequences were identified with MEME from 65 bp sequences upstream of the identified TSS . Sequence logos were generated with WebLogo (http://weblogo.berkley.edu/logo.cgi).

**Table S6. Detection results for surveyed genes in *C. fetus* isolates**

|  | |  |  |  | **Distribution of genes** | | | | | | |
| --- | --- | --- | --- | --- | --- | --- | --- | --- | --- | --- | --- |
| **Strain*^a^*** | | **Source** | **Reference, country*^b^*** | ***galE*** | ***wcbK*** | ***glf*** | ***mat1*** | ***sap*** | ***cas1*** | ***fic3*** | ***fic4*** |
| Cff ATCC 27374 (FR) | | Brain of sheep fetus, type strain | ATCC, FRA | + | + | - | + | B | + | - | - |
| Cff D (F1) | | Bovine | J. Kirpal, GER | + | + | - | + | B | + | - | - |
| Cff B398/2 SK (F5) | | Bovine | AUT | + | + | - | + | B | + | - | - |
| Cff B88 (F6) | | Bovine | E. Hofer, AUT | + | + | - | + | B | + | - | - |
| Cff H88 (F7) | | Bovine | E. Hofer, AUT | + | + | - | + | B | + | - | - |
| Cff S88 (F8) | | Bovine | E. Hofer, AUT | + | + | - | + | B | + | - | - |
| Cff 94/4256 (F9) | | Aborted bovine fetus | S. Hum, AUS | + | + | + | + | AB | - | - | - |
| Cff 107/4172 (F10) | | Aborted bovine placenta | S. Hum, AUS | + | + | - | + | B | + | - | - |
| Cff 133/4369 (F11) | | Aborted bovine fetus | S. Hum, AUS | + | + | - | + | B | - | - | - |
| Cff 12 (F12) | | Human blood | R. Krause, AUT | + | - | + | - | A | + | - | - |
| Cff CCUG 41395 (F13) | | Human | CCUG, SWE | + | - | + | - | A | - | - | - |
| Cff CCUG 43084 (F14) | | Human | CCUG, SWE | + | - | + | - | A | - | - | - |
| Cff CCUG 7473 (F15) | | Human | CCUG, FRA | + | - | + | - | A | - | - | - |
| Cff CCUG 11286 (F16) | | Human blood | CCUG, FRA | + | - | + | - | A | - | - | - |
| Cff CCUG 13315 (F17) | | Human blood | CCUG, FIN | + | - | + | - | A | - | - | - |
| Cff CCUG 17694 (F18) | | Human blood | CCUG, BEL | + | + | - | + | B | + | - | - |
| Cff CCUG 33671 (F19) | | Bull genitals | CCUG, SWE | + | - | + | - | A | - | - | - |
| Cff CCUG 33720 (F20) | | Human peritoneal dialysis fluid | CCUG, SWE | + | - | + | - | A | - | - | - |
| Cff CCUG 39963 (F21) | | Human blood | CCUG, SWE | + | - | + | - | A | - | - | - |
| Cff CCUG 42302 (F22) | | Human blood, diarrhea | CCUG, SWE | + | - | + | - | A | - | - | - |
| Cff CCUG 32676 (F23) | | Human | CCUG, CAN | + | + | - | + | B | + | - | - |
| Cff CCUG 30605 (F24) | | Human blood and gall | CCUG, SWE | + | - | + | - | A | - | - | - |
| Cff L487 (F25) | | Human, diarrhea | G. Feierl, AUT | + | + | - | + | B | + | - | - |
| Cff H97/343 (F27) | | Human, diarrhea | G. Feierl, AUT | + | - | + | - | A | - | - | - |
| Cff H97/292 (F28) | | Human, diarrhea | G. Feierl, AUT | + | - | + | - | A | - | - | - |
| Cff H00/415 (F29) | | Human, diarrhea | G. Feierl, AUT | + | - | + | - | A | - | - | - |
| Cff J 32.844 (F31) | | Human, diarrhea | G. Feierl, AUT | + | - | + | - | A | - | - | - |
| Cff J 35.572 (F33) | | Human, diarrhea | G. Feierl, AUT | + | - | + | - | A | - | - | - |
| Cff f (3208252420) (F34) | | Bull, prepuce | J. Wagenaar, NLD | + | + | - | + | B | + | - | - |
|  | **TABLE S6. –continued** | | | | | | | | | | |
|  | |  |  |  | **Distribution of genes** | | | | | | |
| **Strain*^a^*** | | **Source** | **Reference, country*^b^*** | ***galE*** | ***wcbK*** | ***glf*** | ***mat1*** | ***sap*** | ***cas1*** | ***fic3*** | ***fic4*** |
| Cff 5,5,42 (SZ 107) (F35) | | Ovine | J. Wagenaar, NLD | + | - | + | - | A | - | - | - |
| Cff 98/v445 (F37) | | Bovine | J. Wagenaar, UK | + | + | - | + | B | + | - | - |
| Cff BT36/98 (F38) | | Bovine placenta, abortion | J. Wagenaar, UK | + | - | + | - | A | - | - | - |
| Cff IZ-2149-80 (F39) | | Bull, prepuce | J. Wagenaar, NLD | + | + | - | + | B | + | - | - |
| Cff 122 (F40) | | Ovine | J. Wagenaar, TUR | + | - | + | - | A | - | - | - |
| Cff 89/8/5396 (F41) | |  | J. Wagenaar, ZAF | + | + | - | + | B | + | - | - |
| Cff BT10/98 (F42) | | Ovine | J. Wagenaar, UK | + | - | + | - | A | - | - | - |
| Cff 3754 (D425) (F44) | | Human | USDA, I.Wesley, USA | + | - | + | - | A | + | - | - |
| Cff 82-50 (97-365-1) (F45) | |  | USDA, I.Wesley, USA; J. Wagenaar | + | - | + | - | A | + | - | - |
| Cff 84-32 (F47) | | Bovine vagina | M. Blaser, USA | + | - | + | - | A | + | - | - |
| Cff 82-40 (F48) | | Human blood, renal transplant | M. Blaser, USA | + | - | + | - | A | + | - | - |
| Cfv ATCC 19438 (VR) | | Vaginal mucus of heifer, type strain | ATCC, UK | + | - | - | + | A | - | + | + |
| Cfv 1a (V1) | | Bovine | E. Pohl, GER | + | - | - | + | A | - | - | - |
| Cfv 3 (V3) | | Bovine | J. Kirpal, GER | + | - | - | + | A | - | - | - |
| Cfv G91 (V5) | | Bovine | E. Hofer, AUT | + | - | - | + | A | - | - | - |
| Cfv TH15 (V6) | | Bovine | E. Hofer, AUT | + | - | - | + | A | - | - | - |
| Cfv 80/4172 (V8) | | Bovine | S. Hum, AUS | + | - | - | + | A | - | - | - |
| Cfv 108/4111 (V9) | | Bovine | S. Hum, AUS | + | - | - | + | A | - | - | - |
| Cfv 121/4401 (V10) | | Aborted bovine fetus | S. Hum, AUS | + | - | - | + | A | - | - | - |
| Cfv CCUG 24260 (V11) | | Bovine | CCUG, SWE | + | - | - | + | A | - | - | - |
| Cfv CCUG 33871 (V12) | |  | CCUG, CZE | + | - | - | + | A | - | - | - |
| Cfv CCUG 33872 (V13) | |  | CCUG, CZE | + | - | - | + | A | - | - | - |
| Cfv CCUG 33901 (V14) | |  | CCUG, FRA | + | - | - | + | A | - | - | - |
| Cfv CCUG 33902 (V15) | | Bull, prepuce | CCUG, BEL | + | - | - | + | A | - | - | - |
| Cfv CCUG 35146 (V17) | | Bovine aborted foetus | CCUG, AUS | + | - | - | + | A | - | - | - |
| Cfv CCUG 33936 (V18) | | Cow vagina | CCUG | + | - | - | + | A | - | - | - |
| Cfv CCUG 34394 (V19) | | Bovine | CCUG, ARG | + | - | - | + | A | - | - | - |
| Cfv CCUG 33900 (V20) | | Cow, abortion product | CCUG | + | - | - | + | A | - | - | - |
| Cfv CCUG 34396 (V21) | | Bovine | CCUG, ARG | + | - | - | + | A | - | - | - |
| Cfv NZ 2742-95 (V22) | |  | S. Hum, AUS; provided by A. Burnens | + | - | - | + | A | - | - | - |
|  | **TABLE S6. –continued** | | | | | | | | | | |
|  | |  |  |  | **Distribution of genes** | | | | | | |
| **Strain*^a^*** | | **Source** | **Reference, country*^b^*** | ***galE*** | ***wcbK*** | ***glf*** | ***mat1*** | ***sap*** | ***cas1*** | ***fic3*** | ***fic4*** |
| Cfv NZ 4264-95 (V23) | |  | S. Hum, AUS; provided by A. Burnens | + | - | - | + | A | - | - | - |
| Cfv NZ 4267-95 (V24) | | Bovine | S. Hum, AUS; provided by A. Burnens | + | - | - | + | A | - | - | - |
| Cfv NZ 4266-95 (V25) | | Bovine | S. Hum, AUS; provided by A. Burnens | + | - | - | + | A | - | - | - |
| Cfv NZ 4268 (V26) | | Bovine | S. Hum, AUS; provided by A. Burnens | + | - | - | + | A | - | - | - |
| Cfv NZ 4269-95 (V27) | | Bovine | S. Hum, AUS; provided by A. Burnens | + | - | - | + | A | - | - | - |
| Cfv NZ 4270-95 (V28) | | Bovine | S. Hum, AUS; provided by A. Burnens | + | - | - | + | A | - | - | - |
| Cfv NZ 4272-95 (V29) | | Bovine | S. Hum, AUS; provided by A. Burnens | + | - | - | + | A | - | - | - |
| Cfv NZ 4274-95 (V30) | | Bovine | S. Hum, AUS; provided by A. Burnens | + | - | - | + | A | - | - | - |
| Cfv CCUG 538 (V31) | | Vaginal mucosa of heifer | CCUG | + | - | - | + | A | - | - | - |
| Cfv CCUG 11287 (V32) | | Human blood | CCUG, FRA | + | - | - | + | A | - | - | - |
| Cfv CCUG 7477 (V33) | | Cow abortion product | CCUG | + | - | - | + | A | - | - | - |
| Cfv CCUG 33899 (V37) | | Vaginal mucosa of heifer | CCUG | + | - | - | + | A | - | - | - |
| Cfv CCUG 34335 (V42) | |  | CCUG, URY | + | - | - | + | A | - | - | - |
| Cfv CCUG 34396 (V44) | |  | CCUG | + | - | - | + | A | - | - | - |
| Cfv CCUG 34395 (V45) | | Bovine | CCUG, ARG | + | - | - | + | A | - | - | - |
| Cfv 5,5,21 (V46) | | Bovine | J. Wagenaar, NLD | + | - | - | + | A | - | - | - |
| Cfv 5.5.22 (V47) | | Bovine | J. Wagenaar, NLD | + | - | - | + | A | - | - | - |
| Cfv 44168 (V49) | | Bovine semen | J. Wagenaar, NLD | + | - | - | + | A | - | - | - |
| Cfv 97-v549 (V50) | | Bovine | J. Wagenaar, NLD | + | - | - | + | A | - | - | - |
| Cfv 97-v561 (V51) | | Bovine | J. Wagenaar, NLD | + | - | - | + | A | - | - | - |
| Cfv 97-v566 (V52) | | Bovine | J. Wagenaar, NLD | + | - | - | + | A | - | - | - |
| Cfv 97-v571 (V53) | | Bovine | J. Wagenaar, NLD | + | - | - | + | A | - | - | - |
| Cfv 18156 (V54) | | Bovine semen | J. Wagenaar, NLD | + | - | - | + | A | - | - | - |
| Cfv v311 (V55) | | Bovine | J. Wagenaar, UK | + | - | - | + | A | - | - | - |
| Cfv v315 (V56) | |  | J. Wagenaar, NLD | + | - | - | + | A | - | - | - |
| Cfv LMG 6570 (V57) | |  | BCCM/LMG | + | - | - | + | A | - | - | - |
| Cfv 87-383 (V58) | | Bovine | USDA, L. Tucker, USA; provided by J. Wagenaar | + | - | - | + | A | - | - | - |
| Cfv 3287 (=ADRI-554) (V59) | | Bovine | USDA, I. Wesley (M. Garcia), USA; provided by J. Wagenaar | + | - | - | + | A | - | - | - |
|  | **TABLE S6. –continued** | | | | | | | | | | |
|  | |  |  |  | **Distribution of genes** | | | | | | |
| **Strain*^a^*** | | **Source** | **Reference, country*^b^*** | ***galE*** | ***wcbK*** | ***glf*** | ***mat1*** | ***sap*** | ***cas1*** | ***fic3*** | ***fic4*** |
| Cfv 3280 (=ADRI-502) (V60) | |  | USDA, I. Wesley (M. Garcia), USA; provided by J. Wagenaar | + | - | - | + | A | - | + | + |
| Cfv 3281(=ADRI-510) (V61) | | Bovine | USDA, I.Wesley, USA; provided by J. Wagenaar | + | - | - | + | A | - | - | - |
| Cfv 3288 (=ADRI-555) (V62) | | Bovine | USDA, I. Wesley (M. Garcia), USA; provided by J. Wagenaar | + | - | - | + | A | - | + | - |
| Cfv 8598 (00-695) (V63) | | Bovine | USDA, I.Wesley, USA; provided by J. Wagenaar | + | - | - | + | A | - | - | - |
| Cfv 87-71 (V64) | |  | J. Wagenaar, NLD | + | - | - | + | A | - | - | - |
| Cfv 86-717 (V65) | |  | NHLS, L. Tucker, USA; provided by J. Wagenaar | + | - | - | + | A | - | - | - |
| Cfv 89-630 (V66) | |  | NHLS, L. Tucker, USA; provided by J. Wagenaar | + | - | - | + | A | - | + | - |
| Cfv LMG 93.45 (V67) | | Bovine | J. Wagenaar, BEL | + | - | - | - | A | - | - | - |
| Cfv BT 74/00 (V68) | | Bovine | J. Wagenaar, UK | + | - | - | + | A | - | - | - |
| Cfv 110800-21-2 (V75) | | Bull, prepuce | J. Wagenaar, NLD | + | - | - | - | A | - | - | - |
| Cfv 040900-24a (V76) | | Bull, prepuce | J. Wagenaar, NLD | + | - | - | - | A | - | - | - |
| Cfv 110900-17a (V77) | | Bull, prepuce | J. Wagenaar, NLD | + | - | - | - | A | - | - | - |
| Cfv 511 (V78) | | Bovine semen | J. Wagenaar, HUN | + | - | - | + | A | - | + | - |
| Cfv 515 (V79) | | Bovine semen | J. Wagenaar, NLD | + | - | - | + | A | - | + | - |
| Cfv 84-112 (V81) | | Bovine | M. Blaser, USA | + | - | - | + | A | - | + | + |

*^a^* Abbreviations: Cff, *Campylobacter fetus* subsp. *fetus*; Cfv, *Campylobacter fetus* subsp. *venerealis*; strain designations in our strain collection;

*^b^* Dr. E. Pohl, Deutsche Veterinärmedizinische Gesellschaft, Aulendorf, Germany; Dr. J. Kirpal, Inst. für Mikrobiologie und Tierseuchen der Tierärztlichen Hochschule Hannover, Germany; Dr. E. Hofer, Bundesanstalt für Tierseuchenbekämpfung, Wien, Austria; Dr. R. Krause, Klinische Abteilung f. Pulmonologie und Infektiologie, Universitätsklinik für Innere Medizin, Medizinische Universität, Graz, Austria; Dr. G. Feierl, Institut für Hygiene, Medizinische Universität, Graz, Austria; Dr. J. Wagenaar, Department of Infectious Diseases and Immunology, Utrecht University, The Netherlands; Dr. S. Hum, Camden, Australia; Dr. M. J. Blaser, Department of Medicine and Microbiology, New York School of Medicine, USA; Dr. I. Wesley, USDA, Iowa, USA; Dr. A. Burnens, MCL Laboratories, Dudingen, Switzerland.

Abbreviations: ATCC, American Type Collection; CCUG, Culture Collection, University of Göteborg, Sweden; BCCM/LMG; Bacteria Collection, Laboratorium voor Microbiologie, Universiteit Gent, Belgium; NHLS, National Health Laboratory Service; GER, Germany, AUT, Austria, AUS, Australia; SWE, Sweden; FRA, France; FIN, Finland; BEL, Belgium; CAN, Canada; NLD, The Netherlands; UK, United Kingdom; TUR, Turkey; ZAF, South Africa; CZE, Czech Republic; ARG, Argentina; URY, Uruguay; HUN, Hungary.

**Table S7. Bacterial strains and plasmids used in this study**.

| **Strain** | **Description*^a^*** | **Reference** |
| --- | --- | --- |
| ***C. fetus* subsp*. fetus*** | | |
| ATCC 27374 | Type strain, Nal^r^, SapB | ATCC*^b^* |
| K19 | *Cff* ATCC 27374, *wcbK::P_gatC_*-*aphA-3* | This study |
| 82-40 | Human blood, GenBank AcNo. NC_008599, SapA |  |
| 80-109 | Human blood |  |
| 81-173 | Human cerebrospinal fluid |  |
| 81-200 | Human feces |  |
| 84-88 (1286) | Human blood |  |
| 84-91 (1366) | Human blood |  |
| 84-94 (1367) | Human blood |  |
| 83-88 | Human blood |  |
|  |  |  |
| ***C. fetus* subsp. *venerealis*** | | |
| ATCC 19438 | Type strain, Nal^r^, SapA | ATCC |
| 84-112 | Bovine isolate, genital secretion, Nal^r^ |  |
|  |  |  |
| ***E. coli*** | | |
| DH5α | *endA1 recA1 gyrA96 thi-l hsdR17 supE44 λ^-^ relA1 deoR Δ(lacZYA- argF)- U169 φ80dlacZΔ(M15)* |  |
| S17-*λpir* | Tp^r^ Sm^r^*; recA thi pro hsdR^-^M^+^ RP4:2-Tc::Mu::Km Tn7λpir* |  |
| **Plasmids** |  |  |
| pRYSS1 | *mob_IncP_* |  |
| pRYBM5 | Φ(*P_gatC_-aphA-3*) |  |
| pBlue-mob | *mob_IncP_* in pBluescriptKSII(-) MCS KpnI site | This study |
| pBlue-mob-Km | *P_gatC_-aphA-3* in pBlue-mob NotI/EcoRI site | This study |
| pSW1 | pBlue-mob, *P_gatC_-wcbK::aphA-3,* suicide vector | This study |
| pRYSK12 | Φ(*P_gatC_*-*virD4*), *aphA*-3, *cat*, *mob_IncP_* |  |
| pSW2 | *virD4* of pRYSK12 replaced by *wcK* | This study |

*^a^* Nal^r^, nalidixic acid resistance; Sm^r^, streptomycin resistance; Tp^r^, trimethoprim phenotype; SapA or SapB, sero-/sap-type A or B;

*^b^*ATCC, American Type Culture Collection;

**Table S8. Oligonucleotides used in this study.**

| **#** | **Oligonucleotide*^a^*** | **Sequence*^b^* (5´→ 3´)** | Description and binding site |
| --- | --- | --- | --- |
| 1 | wcbK_screen_f* | GCACAAAGCTTTGTAGGTATAAGC | *wcbK,* (nt 256 to 279)*^c^* |
| 2 | wcbK_screen_r* | CTATAAGCAAATCAACTTCAGCC | *wcbK,* (nt 919 to 987)*^c^* |
| 3 | glf_screen_f* | AACCTGGAGATTATTTTAGCG | *glf,* (nt 530 to 550)*^c^* |
| 4 | glf_screen_r* | ATTTATATTCTCCAAGTCTACC | *glf,* (nt 1054 to 1033)*^c^* |
| 5 | mat1_screen_f* | Attatcctaacaaagatgtaagg | *mat1* (nt 5 to 27)*^c^* |
| 6 | mat1_screen_r* | Taattttctaatgacttttgccg | *mat1* (nt 414 to 392)*^c^* |
| 7 | ACF* | GATAGTCCAGGGGCGGCT | *sapA,* |
| 8 | ACR* | AACCTTATCAAGATCACTAGC | *sapA,* |
| 9 | BCF* | ATTTTATTAAGGAGTTCG | *sapB,* |
| 10 | BCR* | AGCTATAGTATCAGCAACC | *sapB,* |
| 11 | galE_BamHI_fwd* | TAA***GGATCC***ATGAATATCTTGATAACCGGA | *galE,* (nt 1 to 21)*^c^* |
| 12 | galE_PstI_rev* | AAT***CTGCAG***TAGCTATTTTAGTTTCTTCTCC | *galE,* (nt 993 to 975)*^c^* |
| 13 | cas1_probe_F* | AATTATATCACAAGTAGAAGC | *cas1,* (nt 231 to 251)*^c^* |
| 14 | cas1_probe_R* | TAAGACTAAATCTAGCATCG | *cas1,* (nt 661 to 642)*^c^* |
| 15 | Fic3_KpnI_F* | TAA***GGTACC***CATTGATAAAGTTTTGAAATTTTTAG | ICE_84-112, (nt 14,419 to 14,443)*^d^* |
| 16 | Fic3_SalI_R* | TAA***GTCGAC***TTAACATAAGGATAATCCTAA | ICE_84-112, (nt 15,087 to 15,067) |
| 17 | Fic4_KpnI_F* | TAA***GGTACC***CGAATATTTTATAATGTTTCAAGAG | ICE_84-112, (nt 55,519 to 55,542) |
| 18 | Fic4_SalI_R* | TAA***GTCGAC***TTATCTGTTATGCTCCAAATT | ICE_84-112, (nt 56,844 to 56,824) |
| 19 | fw_mob_KpnI | TT***GGTACC***GTTGGCTTGGTTTCATCAGC | *mob IncP^e^* |
| 20 | rv_mob_KpnI | TT***GGTACC***TTCCGTGCATAACCCTGCTT | *mob IncP^e^* |
| 21 | wcbK_EcoRI_f | TAA***GAATTC***GGCAAGCTAAAACAAATTTAGA | 4 nt after *wcbK^e^* |
| 22 | sapB2_HindIII_r | TAA***AAGCTT***CCTTCACCCTCTGTAGGTCTTCC | *sap*-homolog*^e^* |
| 23 | wbbD_SacI_f | TAA***GAGCTC***caatatctatatatg gatgatag | *wbbD^e^* |
| 24 | wcbK_SacII_r | TAA***CCGCGG***GATACAGCTTACAGGATCTGT | *wcbK,* (nt 204 to 184)*^c^* |
| 25 | wcbK_BamHI_f | TAA***GGATCC***ATGAAAACAGTACTTATTACAG | *wcbK,* (nt 1 to 22)*^c^* |
| 26 | wcbK_PstI_r | TTA***CTGCAG***AAACTCAAAACCGTTTTGTATTC | *wcbK,* (nt 1,025 to 1,047)*^c^* |

*^a^* Asterisks indicate primers applied in PCR screens and gene probes for Southern-blots;

*^b^* restriction sites are shown in **bold** and *italics;*

nt, nucleotide; (nt position), in relation to *^c^*nt 1 of the corresponding gene or *^d^*nt 1 of ICE_84-112, *^e^*no appropriate reference sequence available or primer outside of a gene;

**REFERENCES**

1. Gorkiewicz G, Kienesberger S, Schober C, Scheicher SR, Gully C, Zechner R, Zechner EL: **A genomic island defines subspecies-specific virulence features of the host-adapted pathogen *Campylobacter fetus* subsp. *venerealis***. *J Bacteriol* 2010, **192**(2):502-517.

2. Kienesberger S, Schober Trummler C, Fauster A, Lang S, Sprenger H, Gorkiewicz G, Zechner EL: **Interbacterial macromolecular transfer by the *Campylobacter fetus* subsp. *venerealis* type IV secretion system**. *J Bacteriol* 2011, **193**(3):744-758.

3. Sharma CM, Hoffmann S, Darfeuille F, Reignier J, Findeiss S, Sittka A, Chabas S, Reiche K, Hackermuller J, Reinhardt R *et al*: **The primary transcriptome of the major human pathogen *Helicobacter pylori***. *Nature* 2010, **464**(7286):250-255.

4. Perez-Perez GI, Blaser MJ, Bryner JH: **Lipopolysaccharide structures of *Campylobacter fetus* are related to heat-stable serogroups**. *Infect Immun* 1986, **51**(1):209-212.

5. Kienesberger S, Gorkiewicz G, Joainig MM, Scheicher SR, Leitner E, Zechner EL: **Development of experimental genetic tools for *Campylobacter fetus***. *Appl Environ Microbiol* 2007, **73**(14):4619-4630.

6. Darling AE, Mau B, Perna NT: **progressiveMauve: multiple genome alignment with gene gain, loss and rearrangement**. *PLoS One* 2010, **5**(6):e11147.

7. Alikhan NF, Petty NK, Ben Zakour NL, Beatson SA: **BLAST Ring Image Generator (BRIG): simple prokaryote genome comparisons**. *BMC Genomics* 2011, **12**:402.

8. Langille MG, Brinkman FS: **IslandViewer: an integrated interface for computational identification and visualization of genomic islands**. *Bioinformatics* 2009, **25**(5):664-665.

9. Zhou Y, Liang Y, Lynch KH, Dennis JJ, Wishart DS: **PHAST: a fast phage search tool**. *Nucleic Acids Res* 2011, **39**(Web Server issue):W347-352.

10. Grissa I, Vergnaud G, Pourcel C: **CRISPRFinder: a web tool to identify clustered regularly interspaced short palindromic repeats**. *Nucleic Acids Res* 2007, **35**(Web Server issue):W52-57.

11. Schneider TD, Stephens RM: **Sequence logos: a new way to display consensus sequences**. *Nucleic Acids Res* 1990, **18**(20):6097-6100.

12. Gorkiewicz G, Feierl G, Schober C, Dieber F, Kofer J, Zechner R, Zechner EL: **Species-specific identification of campylobacters by partial 16S rRNA gene sequencing**. *J Clin Microbiol* 2003, **41**(6):2537-2546.

13. Hum S, Quinn K, Brunner J, On SL: **Evaluation of a PCR assay for identification and differentiation of *Campylobacter fetus* subspecies**. *Aust Vet J* 1997, **75**(11):827-831.

14. Krause R, Ramschak-Schwarzer S, Gorkiewicz G, Schnedl WJ, Feierl G, Wenisch C, Reisinger EC: **Recurrent septicemia due to *Campylobacter fetus* and *Campylobacter lari* in an immunocompetent patient**. *Infection* 2002, **30**(3):171-174.

15. van Bergen MA, Dingle KE, Maiden MC, Newell DG, van der Graaf-Van Bloois L, van Putten JP, Wagenaar JA: **Clonal nature of *Campylobacter fetus* as defined by multilocus sequence typing**. *J Clin Microbiol* 2005, **43**(12):5888-5898.

16. Woodcock DM, Crowther PJ, Doherty J, Jefferson S, DeCruz E, Noyer-Weidner M, Smith SS, Michael MZ, Graham MW: **Quantitative evaluation of *Escherichia coli* host strains for tolerance to cytosine methylation in plasmid and phage recombinants**. *Nucleic Acids Res* 1989, **17**(9):3469-3478.

17. de Lorenzo V, Timmis KN: **Analysis and construction of stable phenotypes in gram-negative bacteria with Tn5- and Tn10-derived minitransposons**. *Methods Enzymol* 1994, **235**:386-405.

18. Dworkin J, Tummuru MK, Blaser MJ: **Segmental conservation of *sapA* sequences in type B *Campylobacter fetus* cells**. *J Biol Chem* 1995, **270**(25):15093-15101.
